# Elevated adipose inflammation, but reduced hepatic triacylglycerol storage in diet-induced obese *Plin4*^−/−^ mice

**DOI:** 10.1016/j.jbc.2025.111043

**Published:** 2025-12-13

**Authors:** Atanaska Ivanova Doncheva, Ryoko Higa, Prabhat Khanal, Martine Villemo Øksenvåg Ingebrigtsen, Yuchuan Li, Shrikant Kolan, Pratibha Kolan, Ales Kvasnicka, Ingunn Jermstad, Shaista Khan, Bjørn Steen Skålhegg, Svein Olav Kolset, Hilde Nebb, Marit Hjorth, Frode Amador Norheim, Knut Tomas Dalen

**Affiliations:** 1 Department of Nutrition, Institute of Basic Medical Sciences, Faculty of Medicine, University of Oslo, Oslo, Norway; 2 Department of Medical Biochemistry, Oslo University Hospital, Oslo, Norway; 3 Core Facility for Global Metabolomics and Lipidomics, Faculty of Medicine, University of Oslo, Oslo, Norway; 4 The Norwegian Transgenic Center, Institute of Basic Medical Sciences, University of Oslo, Oslo, Norway

**Keywords:** Plin4, lipid droplet, obesity, liver, inflammation, adipose tissue

## Abstract

Plin4 is transcriptionally regulated by peroxisome proliferator–activated receptor gamma and is primarily expressed in white adipose tissue (WAT). We found that expression of Plin4 is elevated in the liver upon prolonged feeding with an obesogenic diet containing saturated fat, fructose, and cholesterol (Western diet). To investigate the functional role of *Plin4* in energy metabolism, we generated *Plin4*^−/−^ mice and assessed effects upon *Plin4* removal in the liver, WAT, and skeletal muscle. Lean *Plin4*^−/−^ mice fed a chow diet had no clear phenotype, except for slightly altered expression of *Plin5* in the heart, liver, and WAT. Obese female *Plin4*^−/−^ mice fed a Western diet had normal metabolic rate, but elevated insulin levels and faster glucose clearance compared to *Plin4*^+/+^ mice. The livers of *Plin4*^−/−^ mice fed a Western diet had normal cholesteryl ester levels, reduced triacylglyceride levels, and reduced expression of endoplasmic reticulum stress markers downstream of PERK. Ovarian WAT of *Plin4*^−/−^ mice fed a Western diet had elevated expression of macrophage markers, higher presence of crown-like structures, but normal adipocyte cell size. In summary, Plin4 deficiency results in subtle systemic effects in diet-obese mice, affecting hepatic lipid storage and adipose inflammation.

Consumption of an energy-dense diet combined with a sedentary lifestyle are strong causal drivers of obesity (1). Obesity is now a major global health concern, as this condition strongly increases the risk of various chronic metabolic disturbances, including metabolic dysfunction-associated steatotic liver disease (MASLD) (2, 3), formerly known as nonalcoholic fatty liver disease (4). Obesity develops with excessive expansion of neutral lipid reservoirs, mainly in the white adipose tissue (WAT) (5). If the capacity of adipose tissue storage is exceeded, lipids will also accumulate ectopically in metabolic tissues like the liver and muscle (6). In mammalian cells, these neutral lipids are mainly deposited as triacylglycerol (TAG) and to a lesser extent as cholesteryl esters (CE). Both of these lipid classes are synthesized in the endoplasmic reticulum (ER) and will often end up in lipid droplets (LDs) that bud off from the cytosolic side of the ER membrane (7, 8). LDs consist of a core of neutral lipids surrounded by a single phospholipid layer with many different embedded proteins with various functional roles. LDs are complex and dynamic organelles with various functions. In addition to their well-known function in energy storage, they also play important roles in biological processes such as membrane biogenesis (9), intracellular signaling, organelle interactions (10), protein degradation (11), and viral particle assembly (12).

Proteins belonging to the perilipin/Plin family are LD-binding proteins important for LD turnover and stabilization of the LD surface (13, 14). The five members of the perilipin family are named *Plin1-5* after their order of discovery (15). The functional roles of Plin1, Plin2, and Plin5 have been extensively investigated in various tissues. These Plins regulate lipolytic degradation of the neutral lipids stored in the core by restricting or facilitating access of lipolytic enzymes to the LD surface (13). It remains to be experimentally verified if Plin3 and Plin4 are involved in the regulation of lipolysis.

Plin4, initially named S3-12 (16), is the least studied member of the perilipin/Plin family. Plin4 was originally cloned and identified as a WAT surface protein, with amino acid sequence similarity to Plin2 (16). The five Plin proteins have structural motifs in common that are limited to this gene family; a N-terminal PAT-domain, a central repeated 33-mer region (that can be subdivided into three 11-mers), and a C-terminal hydrophobic cleft (17, 18). Plin4 deviates from the other Plins with a less conserved PAT domain and a very extended 33-mer repeated region, consisting of ∼29 repeats in Plin4, in contrast to 2 to 3 repeats in the other Plin proteins (16, 17). This region seems to be important for the anchoring of Plin4 to LDs (19). The Plin4 C terminus contains a consensus sequence for geranylgeranylation, as well as a predicted leucine zipper motif that could potentially enable Plin4 to form dimers or other higher-order structures (16). It remains to be determined if these motifs are functional. In support of a possible membrane anchoring, Plin4 is located mainly at the periphery of skeletal muscle fibers (20). However, Plin4 is mostly found intracellularly in various other cell types cultured *in vitro* (19, 21, 22, 23, 24). In lean mice, the expression of Plin4 is mainly restricted to WAT, skeletal muscle, and heart, with lower expression levels in the brain and liver (21, 25). Expression of Plin4 seems highly dependent on peroxisome proliferator-activated receptor gamma (PPARγ) activity, and three PPARγ-binding motifs are functionally mapped in the Plin4 promoter region (25).

In cultured 3T3-L1 adipocytes exposed to high (nonphysiological) levels of fatty acids, Plin4 translocates to nascent TAG-containing LDs (21, 22). Plin4 has consequently been suggested to be involved in the stabilization and/or formation of nascent LDs (21). This is in line with its LD-binding abilities, which suggest that Plin4 binds stronger to TAG than the LD phospholipid layer (19). However, in other cell types exposed to low (physiological) levels of fatty acids, Plin4 seems to preferentially assemble on CE-containing LDs and is rarely observed on TAG-containing LDs (23).

A few studies have investigated the role of Plin4 in adipose TAG accumulation. *Plin4*^−/−^ mice seem to have normal body weight gain and normal adipose tissue mass (26). Two intronic polymorphisms in the human *PLIN4* locus (rs8887 and rs884164) have been reported to modulate adiposity (27), although the same association for rs8887 was not found in another cohort (28). More recently, screening of a large human cohort has linked PLIN4 loss-of-function mutations to increased adiposity with increased risk of developing related health outcomes like type 2 diabetes (29). These associations were, interestingly, more pronounced in women than in men. However, adipose TAG storage seems to be more dependent on the family-related Plin1. Plin1 deficiency leads to loss of adipose lipolytic regulation (30), resulting in lipodystrophy in mice and humans (31, 32). Taken from the above, current evidence suggests that Plin4 is not an important determinant for the formation or storage of adipose TAG-containing LDs.

The first characterization of a *Plin4*^−/−^ mouse model revealed reduced TAG levels in the heart of lean mice, but unaltered TAG levels in several other metabolic tissues analyzed (brown adipose tissue, *soleus* muscle, and liver) (26). This phenotype may be linked to repressed expression of the family-related Plin5 gene, the dominant perilipin that protects TAG stores in the heart (33). To determine the effects of *Plin4* ablation on adiposity and whole-body energy metabolism, we generated a novel floxed *Plin4* mouse model, which was subsequently crossed with CreDeleter mice (34) and backcrossed into C57BL/6N. In this study, we report basic metabolic characterization of *Plin4*^−/−^ mice fed regular chow or a Western diet (WD). We focused our characterization on the liver, as WD feeding strongly induces hepatic Plin4, and WAT, due to the high Plin4 expression in adipocytes.

## Results

### Generation of a floxed Plin4 model

**Figure 1:**
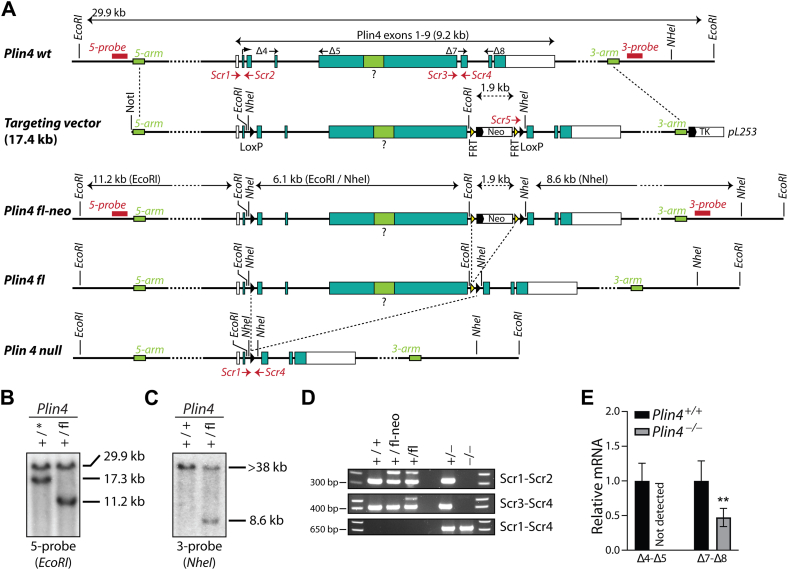
**Generation of *Plin4*^fl^ and *Plin4*^−/−^ mice**. The *Plin4* gene was targeted in embryonic stem (ES) cells using homologous recombination followed by the injection of positive ES cells into blastocysts to disrupt the *Plin4* gene (see Experimental procedures). *A*, a schematic drawing of the WT *Plin4* allele (*Plin4 wt*), the *Plin4* targeting vector, the *Plin4* floxed-_FRT_-Neo-_FRT_ allele (*Plin4 fl-neo*), the *Plin4* floxed allele (*Plin4 fl*), and the *Plin4* null allele (*Plin4 null*). The targeting vector was designed to insert a LoxP site in intron 2 and a LoxP-_FRT_-*Neo*-_FRT_ cassette in intron 6 of the *Plin4* gene. It remains to be determined if the exon encoding the long 33-mer repeated region contains an intronic segment (question mark). Expression of *Flp* enables the removal of the *Neo* cassette and generation of a floxed allele (*Plin4*^*fl*^). *Plin4* floxed-_FRT_-*Neo*-_FRT_ mice were crossed with Cre-Del mice to remove exons 3 to 6 and achieve global deletion of the *Plin4* allele (*Plin4*^−^). Mice were backcrossed for >10 generations into C57BL/6NJr (Janvier Labs) before analysis. The *Scr1-5* arrows mark the binding regions for primers used for PCR genotyping. The Δ4, Δ5, Δ7, and Δ8 arrows mark the binding regions for RT-qPCR primers. *B*, Southern blot analysis of ES cells confirming correct 5-end targeting. *EcoRI*-digested DNA was hybridized with the *Plin4* 5-probe, resulting in the expected fragments of 29.9 kb (*Plin4*^+^ allele) and 11.2 kb (*Plin4*^fl^ allele). Clones with 5-end recombination events in the region between Plin4 exons 3 to 6 lack a LoxP site in intron 2 and give rise to a ∼17.3 kb fragment (*Plin4*∗). *C*, Southern blot analysis of ES cells to confirm correct 3-end targeting. The *NheI*-digested DNA was hybridized with the *Plin4* 3-probe and confirmed to give the expected fragments of >38 kb (*Plin4*^+^ allele) or 8.6 kb (*Plin4*^fl-neo^ allele). *D*, PCR genotyping to confirm the presence of alternative *Plin4* alleles in mice. The primer combinations give rise to the following PCR products: *Plin4*-Scr1 and *Plin4*-Scr2: 232 bp (*Plin4*^+^) and 434 bp (*Plin4*^fl-neo^ or *Plin4*^fl^); *Plin4*-Scr3 and *Plin4*-Scr4: 405 bp (*Plin4*^+^) and 523 bp (*Plin4*^fl^); *Plin4*-Scr5 and *Plin4*-Scr4: 608 bp (*Plin4*^fl-neo^); *Plin4*-Scr1 and *Plin4*-Scr4: 676 bp (*Plin4*^−^). *E*, detection of *Plin4* mRNA transcripts in epididymal white adipose tissue (eWAT) isolated from *Plin4*^+/+^ and *Plin4*^−/−^ mice fed a chow diet. The Δ4-Δ5 primer pair amplifies across the *Plin4* exon4-5 junction and detects WT *Plin4* mRNA only. The Δ7-Δ8 primer pair amplifies across the *Plin4* exon7-8 junction and detects both WT and disrupted *Plin4* mRNAs. Statistical testing was performed with Student *t* test. ∗*p* < 0.05, ∗∗*p* < 0.01 designate difference between *Plin4*^+/+^ and *Plin4*^−/−^ mice. RT-qPCR, reverse transcription quantitative PCR.

To generate mice with a disrupted *Plin4* allele, a Plin4-targeting vector (Fig. 1*A*) was constructed using recombineering (35) and electrophorated into 129-R1 ES cells. Clones with successful homologous recombination events were identified by Southern blotting (Fig. 1, *B* and *C*) and injected into blastocysts to generate chimera containing the altered Plin4 allele. After confirmation of germline transmission, mice were crossed with *Flpe* mice (36) to generate individuals with a floxed *Plin4* allele (*Plin4*^fl^) or with CreDeleter mice (34) to generate individuals with a *Plin4* null allele (*Plin4*^-^). The expected *Flp* and *Cre* recombination events were confirmed by PCR (Fig. 1*D*). The absence of *Plin4* exons 3 to 6 in *Plin4*^−/−^ mice was confirmed by reverse transcription quantitative PCR (RT-qPCR) of epididymal WAT (eWAT) tissue using primers amplifying across the *Plin4* exon 4 to 5 junction (Fig. 1*E*). RT-qPCR with primers amplifying across the *Plin4* exon 7 to 8 junction suggests that the expression of *Plin4*^−/−^ *mRNA* is reduced ∼50% in eWAT compared to the expression of WT *Plin4*^*+/+*^ *mRNA*. The generated *Plin4*^−/−^ mouse model was backcrossed into C57BL/6N for >10 generations prior to the phenotypical characterization.

### Altered *Plin5* expression in various organs of *Plin4*^−/−^ mice

*Plin4*^−/−^ mice were viable at the expected Mendelian inheritance and displayed no clear phenotype in male or female mice on a C57BL/6N background fed a chow diet. Body weights and tissue weights were essentially similar in male *Plin4*^+/+^ and *Plin4*^−/−^ mice, except for a minor reduction in eWAT tissue weight (Table 1). We observed no differences in reproducibility when mating *Plin4*^−/−^ females and *Plin4*^−/−^ males, suggesting that the absence of Plin4 does not affect fertility.

**Table 1:** Body and organ weights of *Plin4*^+/+^ and *Plin4*^−/−^ mice on chow diet

Genotype	*Plin4*^+/+^	*Plin4*^−/−^
15 weeks - males		
Number of animals (n)	8	8
Body weight (g)	29.7 ± 1.9	28 ± 1.7
Liver (g)	1.42 ± 0.	1.35 ± 0.2
Heart (g)	0.120 ± 0.01	0.128 ± 0.01
Kidney (g)	0.35 ± 0.03	0.34 ± 0.05
WAT epidydimal (g)	0.59 ± 0.2	0.25 ± 0.07^a^
WAT subcutaneous (g)	0.36 ± 0.2	0.27 ± 0.3

WAT, white adipose tissue.

Data are presented as means ± SD, n = 8. Statistical testing was performed with Student *t* test.

a *p* < 0.05 designates significant difference between *Plin4*^+/+^ and *Plin4*^−/−^ mice, Student *t* test.

To determine if loss of *Plin4* affected mRNA expression of Plin family members, we measured the expression levels of *Plin1-5* mRNAs in various tissues of 15-weeks-old chow-fed male *Plin4*^+/+^ and *Plin4*^−/−^ mice. Expression of *Plin1-3* mRNAs was essentially similar in all tested tissues of *Plin4*^−/−^ and *Plin4*^+/+^ mice, except for a modestly increased expression of *Plin3* in the soleus muscle of *Plin4*^−/−^ mice (Fig. S1*A*). As expected, *Plin4* mRNA was only detected in *Plin4*^+/+^ mice, confirming that the *Plin4* gene was correctly disrupted in *Plin4*^−/−^ mice. *Plin5* mRNA expression was, however, increased in eWAT and reduced in the muscle *soleus*, heart, and liver of *Plin4*^−/−^ mice compared to *Plin4*^+/+^ mice.

The basal characterization of our novel *Plin4* null model (C57BL/6N background) is essentially in agreement with another *Plin4* null model (C57BL/6J background) by the laboratory of *L*. *Chan* (26), reported to have reduced expression of *Plin5* mRNA in eWAT, liver, and heart (muscle was not assayed). This model differs from ours with opposite effects on *Plin5* mRNA expression in eWAT and stronger reductions in *Plin5* mRNA expression levels in the affected tissues. Hepatic TAG levels were similar in our lean male *Plin4*^+/+^ and *Plin4*^−/−^ mice fed a chow diet (Fig. S1*B*), in agreement with results obtained with the alternative *Plin4* model (26). To conclude, expression of *Plin5* mRNA is only moderately altered in eWAT, soleus muscle, heart, and liver in our *Plin4* null model when fed a regular chow diet.

### Altered hepatic *Plin4* expression in mice fed energy-dense diets or fasted

**Figure 2:**
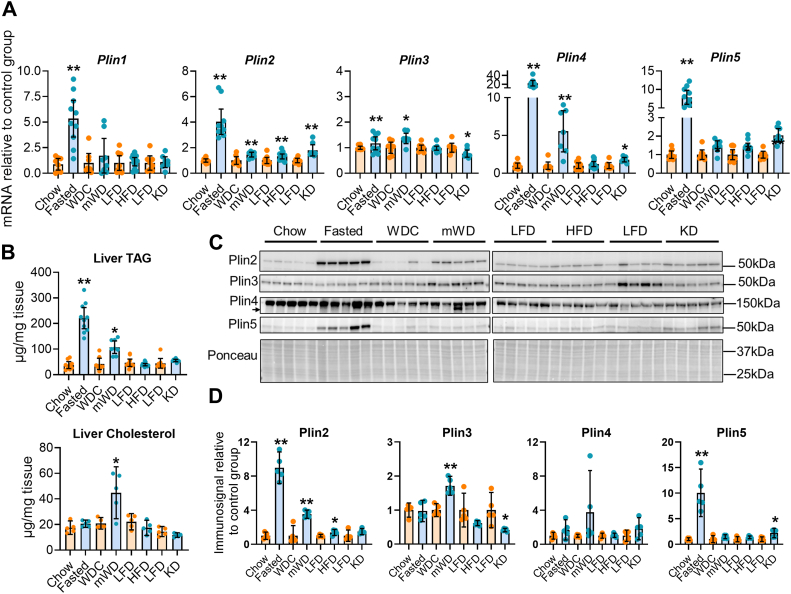
**Perilipin expression and hepatic TAG levels with alternative diets**. Female mice exposed to various dietary treatments: Chow fed (Chow, n = 8) *versus* fasted for 24 h (fasted, n = 10) at 15 weeks of age; Modified western control diet (mWDC, n = 8) *versus* modified western diet (mWD, n = 8) from 8 to 20 weeks of age; low-fat diet (LFD, n = 8) *versus* a high-fat diet (HFD n = 12) from 8 to 20 weeks of age; LFD (n = 8) *versus* a ketogenic diet (KD, n = 8) from 20 to 24 weeks of age. *A*, hepatic *Plin1*, *Plin2*, *Plin3*, *Plin4*, *and Plin5* gene expression levels. Gene expression levels were normalized to expression of *Tbp* and are shown relative to expression levels for the control diet. *B*, liver TAG and cholesterol levels (n = 5). *C*, representative immunoblots of hepatic Plin2-5 protein and Ponceau staining levels in individual mice. *D*, relative quantification of hepatic Plin2-5 immunosignals normalized to Ponceau staining. Data are shown relative to expression of the corresponding control group (n = 5). Data are shown as means ± 95% confidence interval. Statistical testing was performed with Student *t* test for each tissue or diet separately. ∗*p* < 0.05 and ∗∗*p* < 0.01 designate differences in tissue expression between dietary conditions. TAG, triacylglycerol.

Proteins of the perilipin/Plin family are located on the LD surface (13, 14) and have functional roles in cells with accumulated LD. Plin4 is highly expressed in WAT (21, 25), but *Plin4*^−/−^ mice seem to have normal adipose tissue (26). To follow up on the reduced hepatic expression of *Plin5* mRNA in *Plin4*^−/−^ mice, we therefore focused on the liver. To identify conditions where Plin4 may be important for stabilization and lipolytic protection of hepatic LDs, we examined how various dietary regimes affected hepatic expression of the *Plin1-5* genes. Twenty four hours of fasting, a modified Western diet (mWD), a high-fat diet (HFD), and an extreme high-fat/ketogenic diet (KD) were compared against matching control diets. Notably, *Plin4* emerged as the Plin with the most pronounced increase in gene expression in response to either fasting (20-fold) or consumption of the mWD (5-fold) (Fig. 2*A*), with mRNA expression levels associating well with elevations in hepatic TAG (Fig. 2*B*). Hepatic cholesterol levels were increased only with mWD feeding, likely as a result of the higher cholesterol content in this diet (Fig. 2*B*). Expression of *Plin1*, *Plin2*, and *Plin5* mRNAs (Fig. 2*A*) were also elevated by fasting and the various diets, but to a lesser magnitude. Expression of *Plin3* mRNA increased modestly by fasting and mWD feeding, while it was reduced with intake of the KD (Fig. 2*A*). Several of the Plins are posttranslationally regulated and degraded in the absence of LDs (13, 23). We therefore measured hepatic protein expression levels with immunoblotting to correlate Plin-protein expression levels against measured lipids. The expression of Plin2 protein was increased with fasting, mWD, and HFD, while Plin3 was elevated only with mWD (Fig. 2, *C* and *D*). Plin5 protein expression increased with fasting and KD. Plin4 was difficult to detect for all dietary conditions, and faint immunosignals were only observed in mice fed mWD. In conclusion, *Plin4* mRNA levels are not necessarily reflected at the protein level. Although hepatic *Plin4* mRNA levels correlate with hepatic TAG levels, Plin4 protein levels seem more strongly correlated with hepatic cholesterol levels. This observation *in vivo* is in agreement with the Plin4 protein being preferentially associated with CE-containing LDs in cultured cells (23).

### Faster weight gain and faster glucose clearance in *Plin4*^−/−^ mice fed a WD

Given the substantial increase in *Plin4* mRNA expression and detection of the Plin4 protein in the livers with elevated cholesterol, we conducted a prolonged dietary intervention with a Western type of diet (high in fat, sucrose, and cholesterol) to unravel the effects of Plin4 removal. Ten-weeks-old female *Plin4*^+/+^ and *Plin4*^−/−^ mice were fed a low-fat Control diet (CD, 10 kcal% fat) or an obesogenic WD (with high content of cholesterol and fructose (40 kcal% saturated fat, 20 kcal% fructose, and 1.8% cholesterol)) for 45 weeks.

**Figure 3:**
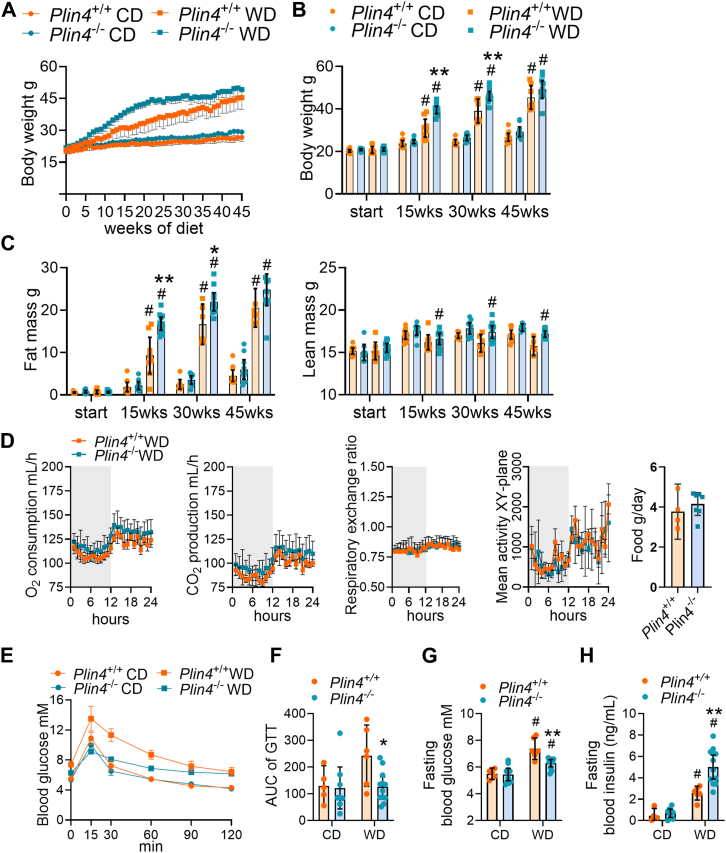
**Body weights and metabolism in *Plin4*^+/+^ and *Plin4*^−/−^ mice fed CD or WD**. Female *Plin4*^+/+^ and *Plin4*^−/−^ mice (10 weeks old) were fed a control diet (CD) or Western diet (WD) for 45 weeks. *Plin4*^+/+^ CD (n = 11), *Plin4*^−/−^ CD (n = 10), *Plin4*^+/+^ WD (n = seven-eighths), and *Plin4*^−/−^ WD (n = 10/11). Mice were euthanized in the morning (8–10 AM) at 55 weeks of age. *A*, body weight curves from the start (10 weeks old) until the end of the dietary intervention (55 weeks old). *B*, total body weights at the beginning (start), 15 weeks, 30 weeks, and 45 weeks of dietary intervention. *C*, fat and lean mass at the beginning (start), 15 weeks, 30 weeks, and 45 weeks of dietary intervention. *D*, metabolic phenotyping of *Plin4*^+/+^ and *Plin4*^−/−^ mice fed WD at 37 weeks of dietary intervention. Oxygen consumption (O_2_ ml/h), carbon dioxide production (CO_2_ ml/h), respiratory exchange ratio, mean activity levels (in the XY-plane) in the light (7 AM – 7 PM) and dark (7 pm-7 am) phases, and daily calorie intake (g/day). *Plin4*^+/+^ WD (n = 4) and *Plin4*^−/−^ WD (n = 7). *E*, plasma glucose (0–120 min) during glucose tolerance testing (GTT) after 30 weeks of dietary intervention. *Plin4*^+/+^ CD (n = 5), *Plin4*^−/−^ CD (n = 8), *Plin4*^+/+^ WD (n = 6), and *Plin4*^−/−^ WD (n = 11). *F*, area under the curve (AUC) calculated blood glucose levels during the GTT (0–120 min). Calculations were made by subtracting the baseline levels (glucose values for 0 min) for all time points. *G*, fasting plasma glucose and (*H*) fasting plasma insulin after 30 weeks of dietary intervention. Statistical testing was performed with two-way ANOVA and Šídák's multiple comparisons test. ∗*p* < 0.05 and ∗∗*p* < 0.01 designate differences between *Plin4*^+/+^ and *Plin4*^−/−^ mice on the same diet. # designates differences between diets for each genotype (*p* < 0.05). Data in graphs are shown as means ± 95% confidence interval.

*Plin4*^+/+^ and *Plin4*^−/−^ mice fed CD had comparable body weights throughout the 45 weeks dietary intervention (Fig. 3, *A* and *B* and Table 2). As expected, mice fed WD increased total body, liver, ovarian WAT (oWAT), and inguinal subcutaneous WAT (subWAT) weights (Fig. 3*B* and Table 2) compared to mice fed CD. Although these weights were comparable between *Plin4*^+/+^ and *Plin4*^−/−^ mice at the end of 45-weeks dietary intervention (Table 2 and Fig. 3*B*), *Plin4*^−/−^ mice fed WD gained body mass and fat mass faster and had increased fat mass compared to *Plin4*^+/+^ mice at 15 and 30 weeks into the dietary intervention (Fig. 3*C*). The lean mass was similar between *Plin4*^+/+^ and *Plin4*^−/−^ mice at all time points during the dietary intervention (Fig. 3*C*), although a small difference by diet was observed for *Plin4*^−/−^ mice.

**Table 2:** Body and organ weights of *Plin4*^+/+^ and *Plin4*^−/−^ mice fed a control diet (CD) or Western diet (WD)

Diet Genotype	CD		WD	
	Plin4+/+	Plin4−/−	Plin4+/+	Plin4−/−
10 weeks – females – before intervention				
Number of animals (n)	12	12	10	11
Body weight (g)	20.3 ± 0.9	20.9 ± 0.73	20.9 ± 2.0	21.0 ± 1.1
55 weeks – females – end of diet intervention				
Number of animals (n)	11	10	7	10
Body weight (g)	26.7 ± 2.7	29.3 ± 3.2	45.4 ± 6.0^a^	49.2 ± 5.7^a^
Liver (g)	1.11 ± 0.14	1.22 ± 0.16	2.8 ± 0.5^a^	3.59 ± 0.65^a^
Heart (g)	0.106 ± 0.008	0.118 ± 0.021	0.125 ± 0.024	0.133 ± 0.008
Kidney (g)	0.25 ± 0.03	0.27 ± 0.02	0.33 ± 0.04	0.37 ± 0.03
oWAT, ovarian (g)	0.7 ± 0.3	0.9 ± 0.4	3.1 ± 1.5^a^	3.7 ± 0.8^a^
subWAT, inguinal (g)	0.5 ± 0.2	0.7 ± 0.3	2.4 ± 0.6^a^	2.8 ± 0.7^a^

CD, control diet, WD, Western diet, high in fat/fructose/cholesterol; WAT, white adipose tissue.

Data are presented as means ± SD. Statistical testing was performed with two-way ANOVA (Tukey’s post-test).

a Designates difference between CD and WD of the same genotype.

To assess potential differences in energy metabolism, *Plin4*^+/+^ and *Plin4*^−/−^ mice fed WD were analyzed with indirect calorimetry. *Plin4*^+/+^ and *Plin4*^−/−^ mice had comparable oxygen (O_2_) consumption, carbon dioxide production (CO_2_), respiratory exchange ratio, physical activity levels, and food consumption (Fig. 3*D*).

We further studied glucose clearance by performing a glucose tolerance test (GTT) of mice fed CD or WD. After injection of glucose (3 g/kg lean mass), blood glucose was substantially increased in *Plin4*^+/+^ mice fed WD, whereas *Plin4*^−/−^ mice fed WD had a faster glucose clearance, more similar to mice fed CD (Fig. 3, *E* and *F*). *Plin4*^+/+^ and *Plin4*^−/−^ mice fed CD had comparable fasting glucose and fasting insulin levels, while *Plin4*^−/−^ mice fed WD had reduced fasting blood glucose (Fig. 3*G*) and increased fasting insulin levels (Fig. 3*H*) compared to *Plin4*^+/+^ mice fed WD.

### Reduced hepatic TAG and Plin2 protein expression in obese *Plin4*^−/−^ mice

**Figure 4:**
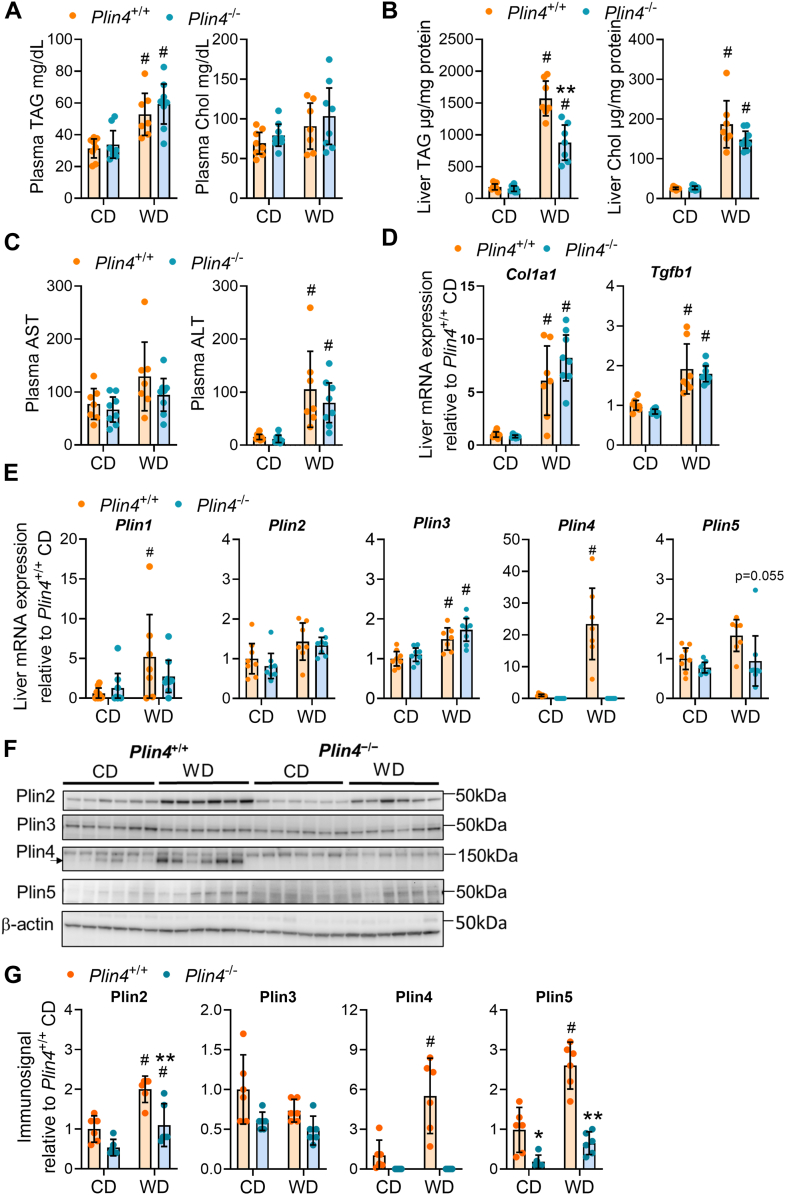
**Lipid abundance and hepatic mRNA expression in *Plin4*^+/+^ and *Plin4*^−/−^ micefed CD or WD**. Analysis of plasma and livers of female *Plin4*^+/+^ and *Plin4*^−/−^ mice fed a control diet (CD) or Western diet (WD) for 45 weeks. *Plin4*^+/+^ CD (n = 8), *Plin4*^−/−^ CD (n = 8), *Plin4*^+/+^ WD (n = 7), and *Plin4*^−/−^ WD (n = 8). *A*, plasma triacylglycerides (TAG) and total cholesterol (Chol) levels. *B*, liver triacylglycerides (TAG) and total cholesterol (Chol) levels. *C*, plasma aspartate aminotransferase (AST) and alanine transaminase (ALT) levels. *D*, gene expression of *Col1a1* and *Tgfb1* mRNAs in the liver relative to expression in *Plin4*^+/+^ mice fed CD. *E*, gene expression of *Plin1*, *Plin2*, *Plin3*, *Plin4*, and *Plin5* mRNAs in the liver relative to expression in *Plin4*^+/+^ mice fed CD. *F*, representative immunoblots of hepatic Plin2-5 and β-actin protein levels in individual mice. *G*, relative quantification of hepatic Plin2-5 immunosignals normalized to β-actin. Data are shown relative to expression in *Plin4*^+/+^ mice fed CD (n = 6). Statistical testing was performed with two-way ANOVA and Šídák's multiple comparisons test. ∗p < 0.05, ∗∗*p* < 0.01 designate differences between *Plin4*^+/+^ and *Plin4*^−/−^ mice on the same diet. # designates differences between diets for each genotype (*p* < 0.05). Data in graphs are shown as means ± 95% confidence interval.

A prolonged feeding with a diet rich in saturated fat and fructose elevates circulating and hepatic TAG, resulting in hepatic lipotoxicity affecting hepatic inflammation and metabolism (37, 38, 39). Mice lacking *Plin2* seem protected against such effects (40, 41), while they seem to worsen in mice lacking *Plin5* (42, 43). Hence, we performed a thorough investigation of liver health in *Plin4*^+/+^ and *Plin4*^−/−^ mice (40, 41, 42, 43). Plasma TAG levels were similarly elevated in *Plin4*^+/+^ and *Plin4*^−/−^ mice receiving WD compared to CD, whereas total cholesterol levels were unaltered (Fig. 4*A*). Hepatic TAG and total cholesterol levels were more strongly increased with WD, but with TAG levels less elevated in *Plin4*^−/−^ mice compared to *Plin4*^+/+^ mice (Fig. 4*B*). Plasma alanine aminotransferase (ALT) levels were increased similarly with WD in *Plin4*^+/+^ and *Plin4*^−/−^ mice, indicative of comparable hepatic stress (Fig. 4*C*), although aspartate transferase (AST) was not significantly increased with WD feeding. Hepatic mRNA expression of the fibrotic and/or inflammatory markers collagen type 1 alpha 1 (*Col1a1*) and transforming growth factor beta 1 (*Tgfb1*) increased similarly in *Plin4*^+/+^ and *Plin4*^−/−^ mice fed WD (Fig. 4*D*), suggesting comparable deteriorating effects on liver health with WD in *Plin4*^+/+^ and *Plin4*^−/−^ mice.

We next determine if the lack of *Plin4* altered hepatic expression of other *Plins*. Expression of hepatic *Plin4 mRNA* was elevated 20-fold with WD in *Plin4*^+/+^ mice (Fig. 4*E*), in contrast to none or subtle effects noted for other *Plin* mRNAs (Fig. 4*E*). Plin2, Plin4, and Plin5 protein levels were increased with WD in *Plin4*^+/+^ mice, while Plin2 and Plin5 protein levels were lower in *Plin4*^−/−^ mice fed WD (Fig. 4, *F* and *G*). Plin3 protein levels were similar across all groups.

### Reduced hepatic TAG and ceramide lipid species in obese *Plin4*^−/−^ mice

**Figure 5:**
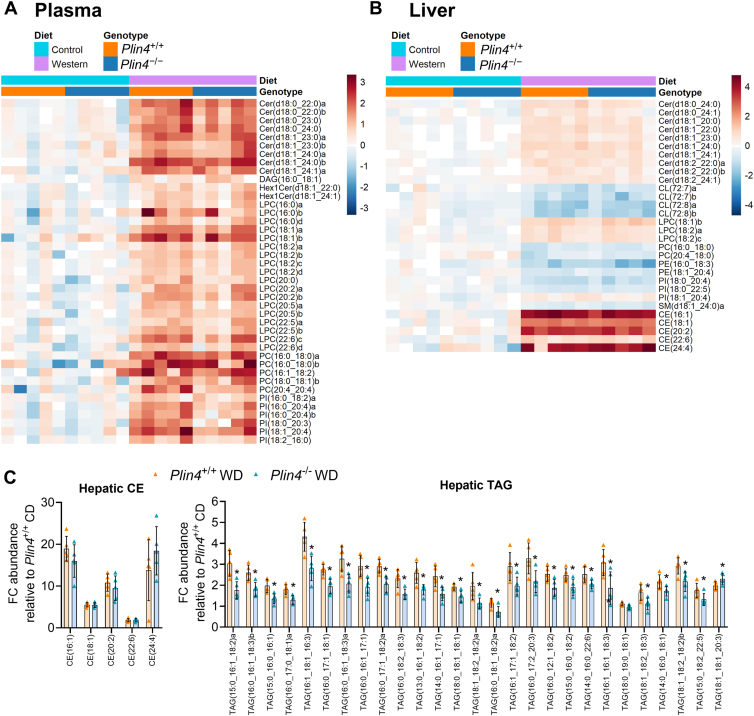
**Untargeted lipidomics analysis of plasma and liver samples of *Plin4*^+/+^ and *Plin4*^−/−^ mice fed CD or WD**. Analysis of plasma and hepatic lipid species in *Plin4*^+/+^ and *Plin4*^−/−^ mice fed a control diet (CD) or Western diet (WD) for 45 weeks. *Plin4*^+/+^ CD (n = 5), *Plin4*^−/−^ CD (n = 5), *Plin4*^+/+^ WD (n = 5), and *Plin4*^−/−^ WD (n = 5). *A*, heatmap showing plasma lipid species present at different levels in CD *versus* WD-fed *Plin4*^+/+^ and *Plin4*^−/−^ mice after adjustments for multiple comparisons (adjusted *p* value < 0.15). Values are scaled as log_2_ fold changes relative to the average expression in the *Plin4*^+/+^ CD group for each lipid species. *B*, heatmap showing hepatic lipid species present at different levels in CD *versus* WD-fed *Plin4*^+/+^ and *Plin4*^−/−^ mice (adjusted *p* value < 0.15). *C*, the abundance of all hepatic CEs detected and hepatic TAG species present at different levels in WD-fed *Plin4*^+/+^*versus Plin4*^−/−^ (adjusted *p* value < 0.15). Data were analyzed with two-sided Welch *t* test and adjusted for multiple comparisons using the Benjamini–Hochberg false discovery rate procedure. ∗ adjusted *p* value < 0.15 designates differences between WD-fed *Plin4*^+/+^ and *Plin4*^−/−^ mice. Data in graphs are shown as means ± SD. CL, cardiolipin; Cer, ceramide; CE, cholesteryl ester; DAG, diacylglycerol; HexlCer, hexosylceramide; LPC, lysophosphatidylcholine; PC, phosphatidylcholine; PE, phosphatidylethanolamine; PI, phosphatidylinositol; SM, sphingomyelin; TAG, triacylglycerol.

Given that hepatic TAG levels were reduced in *Plin4*^−/−^ mice, we performed untargeted lipidomics to determine whether other lipid classes were altered. A total of 262 unique lipid species were detected in the plasma, and 256 were detected in the liver (Table S2, *A*–*B*). Principal component analysis of detected lipid species revealed mostly diet- and minor genotype-dependent differences (results not shown). Among the circulating lipid classes, ceramides (Cer), lysophosphatidylcholine (LPC), phosphatidylcholine, and phosphatidylinositol (PI), several individual lipid species were increased (adjusted *p* value < 0.15) with WD as compared to CD (Fig. 5*A* and Table S2*C*). We next analyzed for genotype differences. No plasma lipid species were different between *Plin4*^−/−^ and *Plin4*^+/+^ mice fed a CD, while two lipid species (LPC(22:0) and PI(18:0_18:2)) were detected at lower levels in *Plin4*^−/−^ mice compared to *Plin4*^+/+^ mice fed WD (Table S2*D*). Other lipid species of these two lipid classes tended to be less abundant, suggesting that plasma levels of these two lipid classes may be somewhat lower in *Plin4*^−/−^ mice than in *Plin4*^+/+^ mice fed WD.

More pronounced differences were observed with diet in the liver. Among the lipid classes diacylglycerol, TAG (Fig. S2), Cer, CE, and LPC, several individual lipid species were increased while cardiolipin was decreased (adjusted *p* value < 0.15) with WD as compared to CD (Fig. 5*B* and Table S2*E*). A few individual lipid species of the lipid classes phosphatidylcholine, phosphatidylethanolamine, PI, and sphingomyelin were also different. When analyzing for genotype differences, four liver lipid species were less abundant in *Plin4*^−/−^ mice compared to *Plin4*^+/+^ mice fed CD (Table S2*F*), but no clear effect on a particular lipid class was found. In contrast, 29 individual lipid species were less abundant in *Plin4*^−/−^ mice compared to *Plin4*^+/+^ mice fed WD. The majority of these lipids belonged to the TAG lipid class, most prominently species with at least one C16 or C18 acyl chains (Fig. 5*C*). Two Cer species (Cer(d18:0_24:0) and Cer(d18:1_23:0)) were also less abundant in *Plin4*^−/−^ mice fed WD, while several others tended to be less abundant. In conclusion, the absence of *Plin4* had little effect on plasma lipids and most hepatic CE levels, but certain hepatic TAG and Cer species were less abundant.

### Altered expression of hepatic ER stress markers in *Plin4*^−/−^ mice fed WD

**Figure 6:**
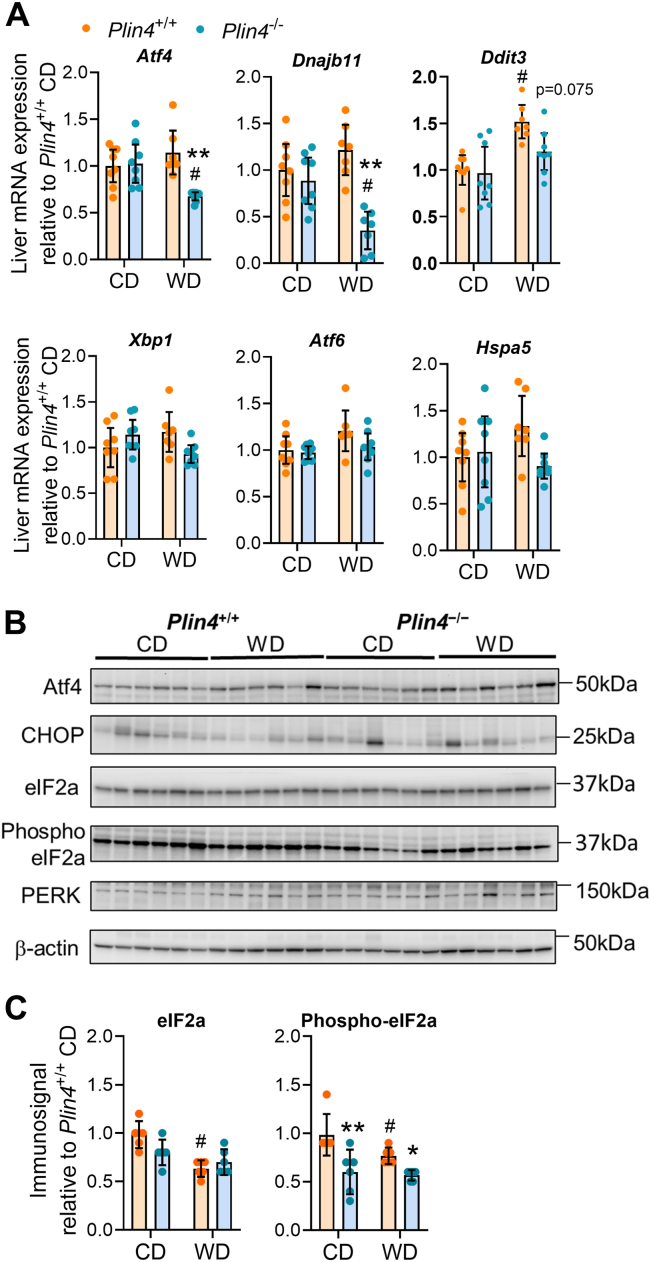
**Expression of ER stress markers in *Plin4*^+/+^ and *Plin4*^−/−^ mice fed CD or WD**. Hepatic gene expression in *Plin4*^+/+^ and *Plin4*^−/−^ mice fed a control diet (CD) or Western diet (WD) for 45 weeks. *Plin4*^+/+^ CD (n = 8), *Plin4*^−/−^ CD (n = 8), *Plin4*^+/+^ WD (n = 7), and *Plin4*^−/−^ WD (n = 8). *A*, gene expression of *Atf4*, *Dnajb1*, *Ddit3*, *Xbp1*, *Atf6*, and *Hspa5* mRNAs in liver relative to expression in *Plin4*^+/+^ mice fed CD. Statistical testing was performed with two-way ANOVA and Šídák's multiple comparisons test. *B*, representative immunoblots of various hepatic ER stress proteins: Atf4, CHOP, eIF2a, phospho-eIF2a, and PERK in individual mice. *C*, relative quantification of hepatic Atf4, CHOP, eIF2a, phospho-eIF2a, and PERK immunosignals normalized to β-actin. Data are shown relative to expression in *Plin4*^+/+^ mice fed CD (n = 6). Statistical testing was performed with two-way ANOVA corrected for multiple comparisons (Benjamini, Krieger, and Yekutieli method). ∗*p* < 0.05, ∗∗*p* < 0.01 designate differences between *Plin4*^+/+^ and *Plin4*^−/−^ mice on the same diet. # designates differences between diets for each genotype (*p* < 0.05). Data in graphs are shown as means ± 95% confidence interval. ER, endoplasmic reticulum; CHOP, c/EBP homologous protein.

Changes in lipid metabolism and LD proteins can influence cellular stress pathways (44). Therefore, we evaluated whether the altered lipid species and reduced Plin2/Plin5 protein levels in *Plin4*^−/−^ mice fed WD coincided with changes in hepatic ER stress. Expression of the ER stress markers activating transcription factor 4 (*Atf4*) and DnaJ heat shock protein family (Hsp40) member B11 (*Dnajb11*) were unchanged by diet in *Plin4*^+/+^ mice but were reduced in the *Plin4*^−/−^ mice fed WD (Fig. 6*A*). *Ddit3* mRNA, encoding for c/EBP homologous protein (CHOP), was increased with WD feeding, but with a tendency to be less expressed in *Plin4*^−/−^ mice (*p* = 0.075) (A). Hepatic expression of *Xbp1*, *Atf6*, and *Hspa5* mRNAs was unaffected by WD and expressed at similar levels in *Plin4*^+/+^ and *Plin4*^−/−^ mice (Fig. 6*A*). Immunoblot analysis showed no significant changes in protein levels for Atf4, CHOP, PERK, and eIF2a, but reduced protein levels of phosphorylated-eIF2a (Fig. 6, *B* and *C*). Under conditions with lipid-mediated ER stress, PERK will phosphorylate eIF2a, which will stimulate transcription of target genes like *Atf4* and *Dnajb11* (45). Altogether, these results suggest that *Plin4*^−/−^ mice fed WD are characterized by reduced hepatic PERK-mediated phosphorylation of eIF2a, resulting in lower transcription of *Atf4* and *Ddit3* mRNAs.

### Lack of Plin4 does not influence hepatic immune cells or lipid metabolism

Next, we performed flow cytometry to determine hepatic inflammation and characterize hepatic immune cell populations in *Plin4*^+/+^ and *Plin4*^−/−^ mice fed WD. Antibodies against CD45 were used to ensure that analyses were restricted to immune cells, combined with specific immune cell markers to distinguish various immune cell populations: macrophages (high F4/80 and intermediate CD11b expression), monocytes (low F4/80 and high CD11b expression), B cells (high CD19 expression), and NK cells (high NK1.1 expression) (Fig. S3*A*). Flow cytometry analysis did not reveal any differences in immune cell populations (Fig. S3*B*) or expression of immune cell markers and inflammatory cytokines (*Cd68*, *Cd79a*, *Klrk1*, *Cd8a*, *Cd4*, *Tnfa*, and *Il1b*) between *Plin4*^+/+^ and *Plin4*^−/−^ mice fed WD (Fig. S3, *C*–*D*).

To investigate if reduced hepatic TAG levels in *Plin4*^−/−^ mice fed WD might be caused by altered lipogenesis, we measured the hepatic expression of key lipogenic genes (Fig. S4, *A*–*B*). Expression of the lipogenic transcription factors Liver X receptor alpha (*LXRa/Nr1h3*), carbohydrate-responsive element-binding protein isoforms (*Mlxipla* and *Mlxiplb*), sterol regulatory element-binding protein 1 isoforms (*Srebf1a* and *Srebf1c*), and its target fatty acid synthase (*Fasn*) were similarly expressed in *Plin4*^+/+^ and *Plin4*^−/−^ mice fed CD or WD. Expression levels of peroxisome proliferator–activated receptor alpha (*Ppara*) and the typical PPAR target gene (*Acox1*) were also similar, suggesting comparable hepatic β-oxidation in *Plin4*^+/+^ and *Plin4*^−/−^ mice. As expected, the high dietary cholesterol content in the WD repressed mRNA expression of *Srebf2* and *Hmgcr* (encoding HMG-CoA reductase) in mice fed WD compared to CD. These mRNAs were similarly expressed in *Plin4*^+/+^ and *Plin4*^−/−^ mice (Fig. S4*C*).

### Unaltered expression of genes involved in glucose metabolism in the muscle of *Plin4*^−/−^ mice

**Figure 7:**
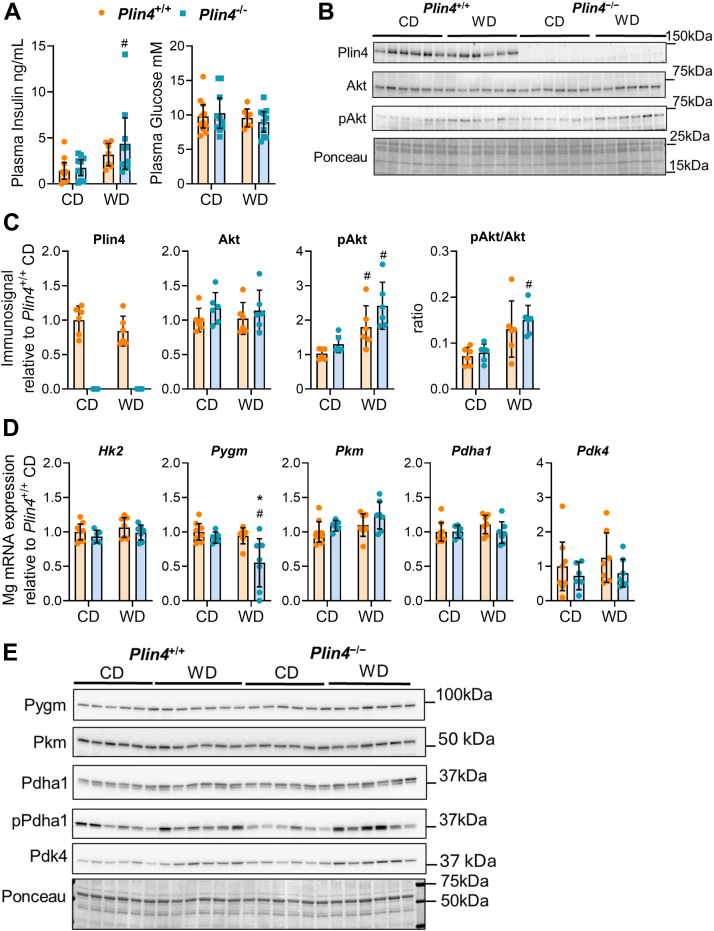
**Expression of genes and proteins involved in glucose metabolism in the muscle *gastrocnemius* (Mg) of *Plin4*^+/+^ and *Plin4*^−/−^ mice fed CD or WD**. *A*, plasma glucose (mM) and insulin (ng/ml) levels at time of euthanasia. *Plin4*^+/+^ CD (n = 11), *Plin4*^−/−^ CD (n = 10), *Plin4*^+/+^ WD (n = 7), and *Plin4*^−/−^ WD (n = 10). *B*, representative immunoblots of Plin4, Akt, pAkt proteins, and Ponceau staining in the muscle gastrocnemius (n = 6). *C*, relative quantification of Plin4, Akt, pAkt, and pAkt/Akt ratio, normalized to Ponceau signals (n = 6). *D*, expression of genes glucose oxidation and glycogen mobilization *Hk2*, *Pygm*, *Pkm*, *Pdha1*, and *Pdk4* in muscle gastrocnemius (n = 8). *E*, representative immunoblots of Pygm, Pkm, Pdha1, pPdha1 proteins, and Ponceau staining in the muscle gastrocnemius (n = 6). Statistical testing was performed with two-way ANOVA corrected for multiple comparisons (Benjamini, Krieger, and Yekutieli method). ∗*p* < 0.05 designates differences between *Plin4*^+/+^ and *Plin4*^−/−^ mice on the same diet. # designates differences between diets for each genotype (*p* < 0.05). Data in graphs are shown as means ± 95% confidence interval. CD, Control diet; pAkt, phosphorylated Akt.

To follow up on the altered glucose clearance in *Plin4*^−/−^ mice fed WD, we focused our attention on genes and proteins related to glucose uptake and metabolism in skeletal muscle by analyzing the *gastrocnemius* muscle, which has a mixed fiber type composition. In contrast to differences observed in the fasted state (Fig. 3, *F* and *G*), plasma glucose levels were similar between all groups at the time of euthanasia in the fed-state (Fig. 7*A*). Immunoblotting revealed similar Plin4 protein levels in *Plin4*^+/+^ mice fed CD and WD and confirmed the absence of Plin4 in the skeletal muscle of *Plin4*^−/−^ mice (Fig. 7, *B* and *C*). Protein expression of the insulin substrate Akt was similar in all groups, while phosphorylated Akt was elevated with WD, with a slightly increased pAkt/Akt ratio in WD-fed compared to CD-fed *Plin4*^−/−^ mice (Fig. 7, *B* and *C*). Expression of mRNAs for hexokinase2 (*Hk2*), essential for trapping of intracellular glucose was the same in all groups, while expression of muscle-associated glycogen phosphorylase (*Pygm*) was slightly reduced in WD-fed *Plin4*^−/−^ mice (Fig. 7*D*). Muscle pyruvate (*Pkm*), pyruvate dehydrogenase alpha 1 (*Pdha1*), and pyruvate dehydrogenase kinase 4 (*Pdk4*), all involved in the mobilization of glucose-derived substrates for the TCA cycle were similarly expressed (Fig. 7*D*). At the protein level, a minor increase in Pdk4 protein levels was observed in WD-fed *Plin4*^−/−^ mice, while Pygm, Pkm, Pdha1, and p-Phda1 protein levels were similar in all groups (Fig. 7, *E* and *F*). In conclusion, these results do not support that the faster glucose clearance in *Plin4*^−/−^ mice is linked to alterations in skeletal muscle insulin signaling, glucose uptake, or metabolism.

### Increased adipose inflammation in *Plin4*^−/−^ mice fed WD

Since Plin4 is highly expressed in WAT (16, 25) and WAT expansion was different in *Plin4*^+/+^ and *Plin4*^−/−^ mice fed WD, we focused our further analysis on WAT in the 55-weeks-old mice. Expression of *Pparg1* and the adipocyte-specific *Pparg2* mRNAs and a well-characterized adipose PPARγ target *Fabp5* were equally expressed in *Plin4*^+/+^ and *Plin4*^−/−^ mice (Fig. S5*A*). TAG levels in oWAT were also similar between all groups (Fig. S5*B*), suggesting that adipocyte differentiation abilities and PPARγ signaling were unaffected by a lack of Plin4. Expression of *Plin1-3* and *Plin5* mRNAs, and Plin1 protein, were essentially similar in all groups in oWAT (Fig. S5, *C*–*E*) and subWAT (Fig. S5*F*). Plin4 mRNA was slightly elevated in oWAT and subWAT in *Plin4*^+/+^ mice fed WD compared to CD.

**Figure 8:**
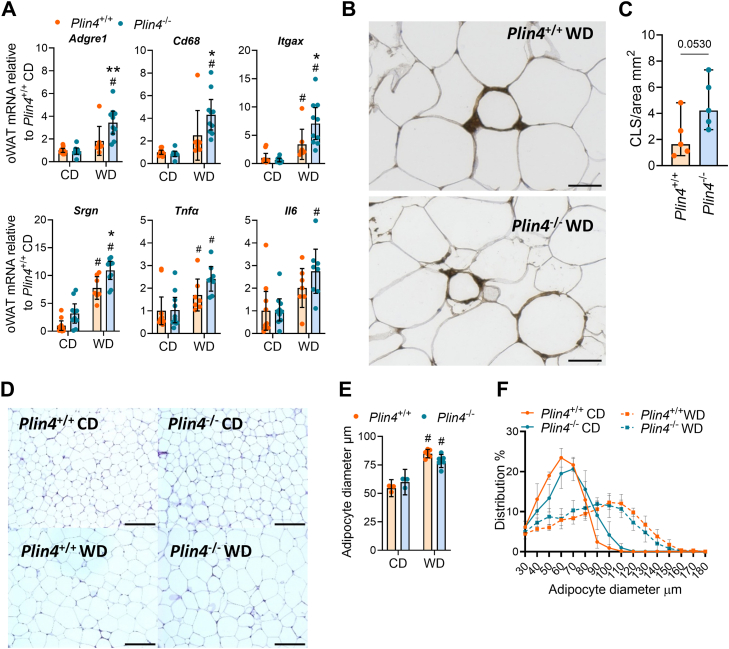
**Adipose inflammation and adipocyte cell size in *Plin4*^+/+^ and *Plin4*^−/−^ micefed CD or WD**. Analysis of adipose tissue of female *Plin4*^+/+^ and *Plin4*^−/−^ mice fed a control diet (CD) or Western diet (WD) for 45 weeks. *A*, expression of *Adgre1*, *Cd68*, *Itgam*, *Itgax*, *Srgn*, *Tnfa*, *and Il6* mRNAs in ovarian WAT (oWAT), relative to expression in *Plin4*^+/+^ mice fed CD. *Plin4*^+/+^ CD (n = 8), *Plin4*^−/−^ CD (n = 8), *Plin4*^+/+^ WD (n = 7), and *Plin4*^−/−^ WD (n = 8). *B*, representative images of Mac2 staining in 5 μm thick oWAT sections from mice fed WD. Scale bar represents 50 μm. *C*, the presence of crown-like structures (CLS) per area (mm^2^) in oWAT of mice fed WD (n = 5). CLS/area mm^2^ represents the mean value per animal, based on the quantification of CLS distributed on three separate sections per animal (representative sections are shown in Fig. S6). Statistical testing was performed with Students *t* test. *D*, representative images of 5 μm thick oWAT sections stained with hematoxylin and eosin. *E*, mean adipocyte diameter based on the quantification of >600 adipocytes in oWAT for each group (CD-fed mice: n = 3, WD-fed mice: n = 6). The scale bar reoresents 200 μm. *F*, adipocyte size distribution based on the quantification of stained adipocytes in oWAT. Statistical testing was performed with two-way ANOVA corrected for multiple comparisons (Benjamini, Krieger, and Yekutieli method). ∗*p* < 0.05 and ∗∗*p* < 0.01 designate differences between *Plin4*^+/+^ and *Plin4*^−/−^ mice on the same diet, # designates differences between diets for each genotype. Data in graphs are shown as means ± 95% confidence interval. WAT, white adipose tissue.

We next compared the expression of ER stress and inflammation markers in oWAT of *Plin4*^+/+^ and *Plin4*^−/−^ mice. Expression of ER-stress markers was similar in the oWAT of *Plin4*^+/+^ and *Plin4*^−/−^ mice (Fig. S5*G*). mRNA expression of typical macrophage markers such as *Adgre1* and *Cd68* were increased more with WD in *Plin4*^−/−^ mice than in *Plin4*^+/+^ mice (Fig. 8*A*). A marker for classically activated macrophages, *Itgax* was expressed at higher levels in *Plin4*^−/−^ mice than in *Plin4*^+/+^ mice fed WD (Fig. 8*A*). The same was observed for *Srgn* (Fig. 8*A*), a cytokine-binding proteoglycan that is positively correlated to the presence of pro-inflammatory macrophages in obese mouse WAT (46), and the inflammatory marker *Il6*. The T-cell markers *Cd4* and *Cd8a* (data not shown) and inflammatory marker *Tnfa* were similarly expressed in *Plin4*^+/+^ and *Plin4*^−/−^ mice (Fig. 8*A*).

The presence of macrophages in WAT is positively correlated with adipocyte inflammation and adipocyte cell size (47). In consistency with signs of increased inflammation, the presence of crown-like structures tended to be increased in the oWAT of *Plin4*^−/−^ mice compared to *Plin4*^+/+^ mice fed WD (Fig. 8, *B* and *C*). Histology of oWAT confirmed that the mean adipocyte cell diameter was increased in WD-fed mice (Fig. 8, *D* and *E* and Fig. S5). Despite differences in the expression of macrophage gene markers and the presence of crown-like structures, we did not observe any differences in the mean adipocyte diameter (Fig. 8*E*) or the cell diameter frequency distribution (Fig. 8*F*) of adipocyte cells in *Plin4*^−/−^ and *Plin4*^+/+^ mice fed WD.

## Discussion

In this study, we characterized a novel congenic *Plin4*-null model on a C57BL/6N background. Using various energy-rich diets, we found that *Plin4* mRNA expression is induced in the liver to a greater magnitude than the other family-related Plins, but the Plin4 protein is difficult to detect in the liver unless the cholesterol content is elevated. When exposed to prolonged feeding with a WD with high content of cholesterol and saturated fat, female *Plin4*^−/−^ mice had higher fat mass, but similar body weights at the end of the 45 weeks dietary intervention. Energy metabolism was unchanged, but *Plin4*^−/−^ mice had faster glucose clearance, perhaps due to higher levels of circulating insulin, judged by higher fasting insulin levels. Additionally, *Plin4*^−/−^ mice had decreased hepatic TAG levels and signs of reduced expression of ER stress markers downstream of PERK. Adipose tissue cell size was unaltered, while expression of inflammatory markers and the presence of crown-like structures were increased. To summarize the phenotypes observed in *Plin4*^−/−^ mice under various dietary conditions, we generated a schematic overview highlighting key findings across tissues and systemic metabolism (Table 3). Overall, the absence of Plin4 in diet-induced obese mice is associated with subtle metabolic differences affecting both adipose tissue and the liver.

**Table 3:** Overview of metabolic effects in *Plin4*^−/−^ mice under different dietary conditions (chow, CD, and WD)

*Plin4*^lin4−/−^ mice compared to *Plin4*^+/+^ on a C57BL/6NRj background			
Tissue/parameter	Chow diet (15 weeks)	Control diet (45 weeks)	Western diet (45 weeks)
Liver			
Perilipins	↓*Plin5* mRNA	↔	↓*Plin5* mRNA and protein ↓Plin2 protein
TAG	↔	↔	↓TAG
Inflammation	NA	↔	↔
ER stress	NA	↓Phospho-eIF2a protein	↓*Atf4* mRNA ↓Phospho-eIF2a protein
Adipose tissue (gonadal)			
Perilipins	↑*Plin5* mRNA	↔	↔
Inflammation	NA	↔	↑*Adgre1*, ↑*Cd68*, ↑*Itgax* ↑*Srgn* mRNAs ↑Crown-like structures
ER stress	NA	↔	↔
Cell size	NA	↔	↔
Muscle gastrocnemius			
Glucose metabolism gene expression	NA	↔	↔
Systemic effects			
Body weight	↔	↔	No difference (gained fat mass faster)
Plasma TAG	NA	↔	↔
Glucose clearance	NA	↔	↑glucose clearance ↑fasting insulin
Metabolic phenotyping	NA	NA	↔

The table summarizes key findings in liver, adipose tissue, muscle, and systemic parameters in *Plin4*^−/−^ mice compared to *Plin4*^+/+^ mice.

Arrows (↑, ↓, and ↔) designate relative increases, decreases, or no alterations, respectively, in gene/protein expression or functional readouts compared to *Plin4*^*+/+*^ controls within the same diet. NA, not analyzed.

A prior characterization of a *Plin4*-null model in a C57BL/6J background did not reveal any adipose phenotype, but a cardiac phenotype with reduced cardiac TAG levels was observed (26). This phenotype was linked to reduced expression of the family-related *Plin5* gene, the dominant Plin that protects TAG stores in the heart (17, 33, 48, 49). Interestingly, *Plin5*^−/−^ mice are protected against cardiac lipid accumulation (33), suggesting that the observed phenotype in this Plin4 model may at least partly be attributed to reduced Plin5 expression. The *Plin4* and *Plin5* genes are positioned adjacent in mammalian genomes. In mice, the 3-end of *Plin5* is oriented 1.7 kb upstream of *Plin4* on the same DNA strand on chromosome 17 (17). In the lack of a clear molecular explanation for the repressed *Plin5* expression in *Plin4*^−/−^ mice, *Chen et al*. speculated that genetic manipulation of the *Plin4* locus may have unintentionally influenced *Plin5* expression. Their *Plin4*^−/−^ model disrupted the *Plin4* gene by replacing the majority of the *Plin4* coding region (exons 2–9) with a selection cassette (26), which potentially can affect the transcription from nearby loci (50). In our *Plin4* null model, the genetic segments encoding the 11-mer repeats (exons 5–6) have been removed without inserting a selection cassette. Still, in partial agreement with the first model (26), the expression of Plin5 was slightly altered in several tissues. Male *Plin4*^−/−^ mice fed a standard chow diet had reduced *Plin5* mRNA expression in the heart, liver, and muscle *soleus*, but increased expression levels in eWAT. Hence, disruption of *Plin4* seems to have tissue-specific effects on *Plin5* expression levels. We have previously mapped various cis-regulatory PPAR-binding elements in the promoter region of the Plin genes. *Plin4* is regulated by PPARγ in adipose tissue (25), while *Plin5* is regulated by PPARα in the liver (17) and PPARδ in the muscle (51). All of the cis-regulatory PPAR response elements elements that are functionally verified to be involved in transcriptional regulation of the *Plin4* and *Plin5* genes are located distant from the *Plin4* genomic regions being manipulated in both *Plin4*^−/−^ models. If other uncharacterized genomic cis-regultory elements are lost in the null models, resulting in *Plin5* down and up regulation in various tissues, remains to be examined. Of note, *Plin5*^−/−^ mice are protected against lipid accumulation in the heart (33), but without affecting *Plin4* mRNA and protein expression levels (43). This argues against a simplistic transcriptionally mediated model where Plin4 and Plin5 proteins affect the expression of their counterparts. Such a compensatory regulation is not uncommon among Plin proteins. For example, Plin2 is highly expressed in both liver and adrenals. In the liver of fasted *Plin2*^−/−^ mice, the expression levels of Plin5, and to a lesser extent Plin3, are elevated (52), while the expression levels of Plin1 and Plin3 are elevated in the adrenals (53). It is generally believed that family-related Plin proteins can partially compensate for each other and bind to LD surfaces during conditions of high-lipid loading and LD expansion. However, each Plin is also likely to have unique function(s) that may lead to secondary effects on LD metabolism, lipid flux, and energy metabolism upon their removal.

Obesity increases the risk for MASLD (2) and insulin resistance (54), partly driven by dysregulated fatty acid trafficking and storage in obese WAT, leading to fatty acids being stored in nonadipose tissue organs (ectopic fat deposition) like the liver. Plin4 is weakly expressed in the liver in lean mice where hepatic lipid levels are low (25, 26) but is induced in expression by various dietary conditions that elevates hepatic lipid levels and may therefore be important for liver function in steatotic liver. We observed that *Plin4* mRNA, but not necessarily Plin4 protein levels, is strongly induced in the liver upon fasting or in obese mice fed energy-rich diets. In addition, *Plin4* mRNA expression levels seem to correlate well with hepatic TAG levels. Elevation of hepatic TAG, inflammation, and in some cases fibrosis are key components of MASLD (55). To determine the role of Plin4 in MASLD, we performed a comprehensive lipid analysis of the livers of *Plin4*^+/+^ and *Plin4*^−/−^ mice fed CD or WD for 45 weeks. Hepatic TAG and CE levels were highly induced by WD, but while TAG levels were reduced in *Plin4*^−/−^ mice fed a WD diet, CE levels were similar. Untargeted lipidomics analysis revealed that the absence of *Plin4* predominantly affected a large repertoire of hepatic TAG species and a few Cer species, in contrast to modest alterations in plasma lipid profiles. The population of hepatic immune cells, as well as the expression of inflammation (*Tgfb*) and fibrotic (*Col1a1*) markers were similar. Phosphorylated eIF2 and expression of *Atf4* and *Ddit3* mRNAs were repressed in *Plin4*^−/−^ mice fed a WD, suggesting that PERK signaling may be reduced. Elevated hepatic TAG may disturb lipid metabolism and mitochondrial function, culminating in the production of reactive oxygen species and ER stress–induced apoptosis (56). The role of Plin4 in MASLD can therefore be interpreted differently; lack of Plin4 may be seen as protective by reducing hepatic TAG levels or alternatively, Plin4 may be seen as not protective given that the expression of inflammatory and fibrotic markers is unaffected, despite reduced TAG levels and signs of reduced ER stress. Plin4 may also play an indirect role in MASLD due to systemic secondary effects caused by alterations in other tissues, such as WAT. All considered, our analyses point to a hepatic defect in lipid handling in obese *Plin4*^−/−^ mice under metabolic stress.

We observed that adipose cell sizes were comparable in obese *Plin4*^−/−^ mice and *Plin4*^+/+^ mice. Although Plin4 does not seem to be important for WAT differentiation and adipose TAG accumulation *per se*, it may affect adipocytes and/or adipose tissue function and signaling. Plin4 has been observed on nascent LDs in cultured 3T3-L1 adipocytes, but importantly, under experimental conditions where nonphysiologically high levels of fatty acids (1.8 mM) were used (21, 22). In other cell types exposed to physiologically relevant levels of fatty acids (0.05–0.1 mM), Plin4 binds preferentially to LDs containing CE and is rarely observed on LDs containing TAG (23). In this study, we observed that hepatic Plin4 protein content seems to depend on elevated hepatic cholesterol and/or CE (WD diet feeding), albeit lack of Plin4 predominantly reduces TAG stores without affecting CE stores. A possible explanation for these puzzling observations is that Plin4 perhaps preferentially bind to LDs containing CE, but without protecting against lipolysis and LD degradation. Alternatively, other lipid species that are elevated with WD are needed for stabilization of the Plin4 protein. Regardless, the strong induction of both *Plin4* mRNA and TAG levels in a fasted liver without noticeable detection of Plin4 protein suggests that Plin4 protein levels are unaffected by LDs containing TAG. Earlier observations in WAT also suggest the same, since *Plin4*^−/−^ fibroblasts cultured *in vitro* differentiate normally into adipose cells with no effect on TAG stores, and both lipolysis and fat mass accumulation *in vivo* seem normal in *Plin4*^−/−^ mice (ref (26) and this study). A detailed analysis of the LD-binding preferences for Plin4 will be needed to clarify the above. Available data suggest that the functional role of Plin4 is likely to involve its ability to bind to LD surfaces, but not to be directly related to the protection of TAG reservoirs.

Obesity is an important driver of adipose inflammation (57), and the quantity of macrophages and formation of crown-like structures in WAT is positively correlated with WAT expansion (47). *Plin4*^−/−^ mice fed WD had similar body weight as compared to *Plin4*^+/+^ mice after 45 weeks of feeding, but seemed to gain fat faster. Existing data regarding potential effects on body weights upon loss of *Plin4* is limited. Male *Plin4*^−/−^ mice are reported to have normal body weights and adipose tissue weights at 13 weeks of age (26). In our study, we observed unaltered body weights for chow-fed male mice at 15 weeks of age and female mice at 10 weeks of age prior to initiation of the dietary intervention. A possible diet-dependent effect on body weight in female *Plin4*^−/−^ mice must be examined more carefully. However, the faster weight gain in *Plin4*^−/−^ mice fed a WD is in line with increased body weights in women with loss-of-function mutations in the *Plin4* gene (29). In our WD intervention in female mice, it is possible that redirection of lipids to WAT may have protected the livers of *Plin4*^−/−^ mice at the earlier stages of the intervention and prevented the development of obesity-related metabolic complications in that organ. Contrarily, a more rapid expansion of the adipose tissue in *Plin4*^−/−^ mice may have triggered adipose inflammation in oWAT, manifested as increased mRNA expression of macrophage markers and a higher content of crown-like structures. While energy metabolism seems to be unaffected in this intermit period, GTT revealed higher fasting insulin levels but lower basal fasting glucose and faster glucose clearance in *Plin4*^−/−^ mice, despite higher adipose tissue mass. Despite faster glucose clearance in *Plin4*^−/−^ mice, the expression of key genes in glucose uptake and glucose metabolism in skeletal muscle remained unchanged when measured at the end of the intervention. These findings imply that altered glucose clearance in *Plin4*^−/−^ mice is related to hepatic or adipose tissue adaptations. Analysis of glucose homeostasis using tissue-specific Plin4 null models will be needed for definitive conclusions.

In summary, we have characterized a novel Plin4 null model where reduced expression of Plin5 in various tissues is less pronounced. Our characterization of this model provides new clues to the functional role of the fourthly discovered perilipin family member (16), now renamed perilipin4/Plin4. Loss of Plin4 in diet-induced obese mice leads to slightly altered expression of Plin5 in various organs, and subtle metabolic effects, including reduced hepatic TAG, signs of altered hepatic ER stress, elevated adipose inflammation, and systemic alterations in glucose clearance.

## Experimental procedures

### Materials

Plasticware was obtained from Costar (Merck) and Sarstedt. Reagents for ES cell culturing, bacterial culturing (LB-medium), and primers were obtained from Sigma-Aldrich. Materials for real-time quantitative PCR were obtained from Bio-Rad Laboratories and Applied Biosystems (Thermo Fisher Scientific). Paraformaldehyde (#19210) was obtained from Electron Microscopy Sciences.

### Cloning of the Plin4 targeting vector

The *Plin4*-Flox-Neo targeting vector was generated using recombineering (35). Briefly, a prescreened BAC clone obtained from AB2.2 ES cells (strain: 129S7/SvEvBrd-Hprtb-m2, clone: #bMQ-335E9; Sanger (34)) was electroporated into *Sw102 Escherichia coli*. Thereafter, a 17.4 kb segment spanning the whole *Plin4* locus was cloned using gap-repair into a pL253 vector containing a thymidine kinase cassette, consisting of the HSV-*tk* gene expressed by the MC1 promoter (*Mc1tk*), to generate the pL253-*Plin4* vector. Two mini-cassettes used to insert LoxP sites were constructed in pPCR-Script vectors (Agilent). Mini-cassettes were digested out with NotI/SalI, purified on an agarose gel, and sequentially co-electroporated (∼200 ng) with the pL253-Plin4 vector (∼5 pg) into *Sw102* bacteria. The obtained *Plin4* targeting vector (*Plin4*-Flox-Neo, see Fig. 1) contained a LoxP site inserted in intron 2 and a Neomycin-selection cassette flanked by FRT sites/LoxP-site (FRT-Neo-FRT-LoxP) inserted in intron 6. Primers used for all cloning steps are listed in Table S1.

### Animal ethics and housing conditions

All experimental use of animals was approved and registered by the Norwegian Animal Research Authority (Mattilsynet, approvals FOTS ids: #912, #3165, #6922, #6305, #10901, #10902, and #24912). Experiments conformed to ARRIVE guidelines 2.0, national legislations (FOR-2015-06-18-761), and ethical guidelines in Directive 2010/63/EU of the European Parliament on the protection of animals used for scientific purposes. Mice were housed in individually ventilated cages (#GM500, Techniplast) with 75 air changes per hour in a room with a stable light/dark cycle (7 AM to 7 PM), with 55 ± 5% relative humidity at 22 ± 2 °C. Animals had free access to sterilized water and rodent chow diet. The presence of pathogens was monitored quarterly in accordance with FELASA (Federation of European Laboratory Animal Science Associations) recommendations. Animals used in the dietary interventions were specific pathogen-free according to FELASA recommendations (specific pathogen-free status).

### Generation of the floxed *Plin4* mouse model

Linearized (Not I digested) *Plin4*-Flox-Neo vector was electroporated into 129-R1 ES cells. ES cell clones with the desired homologous recombination event were identified by Southern blotting using Turbo blotter (Schleicher & Schuell, Sigma-Aldrich) followed by hybridization of membranes with [α-^32^P]dCTP (PerkinElmer) radiolabeled probes generated with the Megaprime DNA labeling System (Amersham Biosciences). Targeting efficiency was ∼20% (out of 117 screened ES clones, 43 clones had successfully incorporated a LoxP-Neo cassette in exon 6 (3-end positive), and around half of these (23 clones) had also incorporated a LoxP site in exon2 (5-end and 3-end positive)). Primers used for the amplification of genome segments used as templates for Southern probes are listed in Table S1.

Selected positive ES cells were micro-injected into blastocysts derived from C57BL/6N females and transferred to pseudo-pregnant RjOrl:SWISS recipients (Janvier Labs). Chimeric offspring were mated with C57BL/6N mice. Germline transmission was confirmed by PCR. Offspring with a modified *Plin4* allele (*Plin4*^fl-Neo^) were mated with *FLPe* mice (36) to remove the Neo-selection cassette and generate mice with a floxed *Plin4* allele (*Plin4*^fl^). *Plin4*^fl-Neo^ mice were mated with Cre Deleter mice, expressing an X-linked Cre recombinase (58), to generate mice with a total deletion of *Plin4* (*Plin4*^−/−^) mice. The expected deletion of the Neomycin-cassette and *Plin4* exons 3 to 6 in *Plin4*^fl^ and *Plin4*^−/−^ mice, respectively, was confirmed by genotyping (using PCR) and detection of alternative *Plin4* transcripts (using RT-qPCR) (see Fig. 1). Primers used for genotyping and RT-qPCR analysis are listed in S-1 and S-2.

### Animal groups and diet interventions

#### Dietary interventions in WT mice

The female C57BL/6NRj mice (Janvier Labs) used were derived from internal breeding (F_2_ or F_3_ litters). All mice had *ad libitum* access to a chow diet (#2018S; 58 E% carbohydrate, 18 E% fat, 24 E%; Teklad Global 18% Protein Rodent Diet, Envigo) before altered diets were given. *Fasting intervention*: Mice at 15 weeks of age remained on a chow diet or were fasted for 24 h. *mWD intervention*: Mice were fed with either a modified western control diet containing 60 E% carbohydrate, 20 E% fat, 20 E% protein (#D17081902i, Research Diets) or a mWD containing 1% cholesterol, 40 E% carbohydrate, 40 E% fat, 20 E% protein (#D17081901i; Research Diets) from 8 weeks of age until 20 weeks of age (12 weeks dietary intervention). HFD *intervention*: Mice were fed either a low-fat diet containing 70 E% carbohydrate, 10 E% fat, 20 E% protein (#D12450Ji; Research Diets) or a HFD containing 20 E% carbohydrate, 60 E% fat, 20 E% protein (#D12492i; Research Diets) from 8 weeks of age until 20 weeks of age (12 weeks dietary intervention). KD *intervention*: Mice were fed low-fat diet (#D12450Ji) or a KD containing 0.1 E% carbohydrate, 80 E% fat, 20 E% protein (#D03022101i; Research Diets) from ∼20 weeks of age until 24 weeks of age (4 weeks dietary intervention). A total of 73 mice were used in these dietary interventions.

#### Studies in Plin4*^−/−^mice*

The *Plin4*^−/−^ mouse model was back-crossed into C57BL/6NRj (Janvier Labs) for >10 generations prior to experiments. *Plin4*^*−/−*^ *mice on chow diet*: Male *Plin4*^+/+^ and *Plin4*^−/−^ mice (n = 8, n = 8) were housed with free access to rodent chow diet containing 58 E% carbohydrate, 18 E% fat, 24 E% (#2018S; Teklad Global 18% Protein Rodent Diet, Envigo). The 15-weeks-old male mice were euthanized in the morning (08–10 AM). Body and tissue weights were measured at the time of euthanasia, and dissected tissues were immediately snap-frozen in liquid nitrogen. All animals were included in all analyses. *Plin4*^*−/−*^ *mice on WD*: Female *Plin4*^+/+^ and *Plin4*^−/−^ mice were housed with free access to a rodent chow diet containing 62 E% carbohydrate, 11 E% fat, 27 E% protein (#RM3A; Special Diet Services). At 10 weeks of age, mice were randomly assigned to either a low-fat CD consisting of 70 E% carbohydrate, 10 E% fat, 20 E% protein (#D12450Ji) or a standard WD high in saturated fat, fructose, and 1.8% cholesterol, with 40 E% carbohydrate, 40 E% fat, 20 E% protein (#D09100310i; Research Diets). Mice were maintained on these diets until 55 weeks of age. Body weights were determined weekly. Body composition of mice was determined with noninvasive magnetic resonance imaging (Minispec LF90II, Bruker) at 10, 25, 40, and 55 weeks of age (0, 15, 30, and 45 weeks on special diets). A total of 45 *Plin4*^+/+^ and *Plin4*^−/−^ mice were included in the study. Seven animals (1–3 animals per group) were removed from the study due to wounds/untreatable dermatitis, sickness, or unexpected death. These events were not linked to a particular genotype or diet. For certain analyses, representative subsets of animals were selected prior to analysis, with all collected data included in the analysis. The number of animals or samples included in each analysis is shown in the figure legends.

### Metabolic phenotyping

#### Indirect calorimetry

Indirect calorimetric measurement of oxygen consumption and carbon dioxide production was performed at 47 weeks of age. Mice were accustomed to single housing in metabolic cages (Phenomaster, TSE Systems) for 72 h before data collection for the next 48 h. Gas flow was set at 0.42 L/min. Gas measurements (mean values for 10 s) were performed in 20 min intervals per cage. The average of the three measurements per hour was used to calculate gas values per cage per hour. Physical activity was recorded as movements in the XY plane.

#### GTT

40-weeks-old mice were withdrawn from food for 6 h (from 7 AM until 1 PM), before i.p injection of 3 g/kg lean mass of D-(+)-Glucose (#G7021, Sigma) of a stock solution containing 40% glucose solved in saline. Blood was collected from the tail prior to (0 min) and 15, 30, 60, 90, and 120 min after glucose injection. Blood glucose levels were measured directly in whole blood using a glucose meter (FreeStyle Precision Neo, Abbot). Insulin concentrations in plasma were measured with ELISA (#90080, Crystal Chem) following the manufacturer's instructions.

### Hepatic immune cell preparation and flow cytometry

Mice were sedated by isoflurane inhalation and blood was collected by cardiac puncture with an EDTA-coated syringe. Mice were subsequently sacrificed with cervical dislocation. The peritoneal cavity was opened, and the portal vein was cannulated with a 27G needle. The liver was perfused with PBS using a peristaltic pump (flow rate at 6 ml/min) until pale. The perfused liver was dissected from the body cavity and the liver weight was recorded. The large liver lobe of each mouse was used for the preparation of the immune cell suspension (around 0.6 g), while the remaining liver was snap-frozen for other downstream analyses.

Perfused liver tissue was placed in 15 ml of DMEM (#D5671, Sigma) supplemented with 100 mM L-glutamine (#G7513, Sigma), 10% (v/v) fetal bovine serum (#F7524, Sigma), 0.75 mg/ml collagenase A (#C5138, Sigma), and 50 μg/ml DNase I (#10104159001, Sigma). Hepatic immune cells were isolated according to Daemen *et al*. (59). Prior to flow cytometry analysis, the immune cell suspension was diluted to 3 x 10^6^ cells/ml in fluorescent activated cell sorting (FACS) buffer (PBS supplemented with 2.5% (w/v) bovine serum albumine and 2 mM EDTA). Cells were spun down at 650g for 4 min. The cell pellet was resuspended in 50 μl Fc block-buffer containing 0.5 μg/ml CD16/CD32 monoclonal antibody (#14-0161-85, Thermo Fisher Scientific) in FACS buffer and incubated on ice for 30 min in the dark. A mixture of the following fluorescent conjugated primary antibodies was then added to the cell suspension: anti-mouse CD45 clone 30-F11, #48-0451-82; anti-mouse F4/80 clone BM8, #69-4801-82; anti-mouse CD11b clone M1/70, #53-0112-82; anti-mouse CD19 clone eBio1D3, #12-0193-82; anti-mouse NK1.1 clone PK136, #17-5941-82 (all from Thermo Fisher Scientific). The cell suspension (a final volume of 100 μl) was subsequently incubated on ice for 45 min in the dark. Cells were centrifuged and washed once with 300 μl FACS buffer, then centrifuged again and resuspended in 500 μl FACS buffer. Flow cytometry was performed using a BD FACSCanto II Flow Cytometer (BD Biosciences). Analysis of the data and generation of graphs were performed in FlowJo V10 Software (BD Biosciences).

### Isolation of total RNA

Total RNA from the liver, heart, brown adipose tissue, and muscle was isolated with a NucleoSpin RNA kit (Macherey-Nagel, GmbH & Co. KG) using a modified protocol to increase RNA yield and purity (53). Briefly, a phenol:chloroform:isoamylalcohol extraction step and addition of a high salt solution to the aqueous phase were performed before column loading to increase RNA yield. Samples were subsequently loaded on the silica column for RNA purification and DNase treatment according to the manufacturer’s instructions. Total RNA from eWAT, oWAT, and subWAT was isolated with Trizol (#15596026, Thermo Fisher Scientific) and purified on NucleoSpin as described in (46). RNA was eluted in RNase-free water. RNA concentration and integrity (260/280 ratio) were determined using a NanoDrop ND-1000 Spectrophotometer (Thermo Fisher Scientific). Isolated RNA was stored at - 80 °C until use.

### Reverse transcription quantitative PCR

#### Reverse transcription of RNA

Total RNA (12.5 ng/μl) was reversely transcribed with random hexamers and the High Capacity cDNA Reverse Transcription Kit (#4368814, Thermo Fisher Scientific) on an ep Gradient S Eppendorf Mastercycler (Eppendorf AG) with the following settings: 25 °C for 10 min, 37 °C for 120 min, 85 °C for 5 min, and 4 °C on hold.

#### qPCR with fluorescent intercalating dye

Gene-specific regions were amplified from cDNA (5–10 ng/μl) with assay primers (200 nmol/L each) and Bio-Rad SsoAdvanced Universal SYBR Green Supermix (10 μl reaction, 95 °C for 3 min, followed by 40 cycles of 95 °C for 10 s and 60 °C for 20 s) on a CFX96 Touch PCR instrument (Bio-Rad). Assay primers were designed with Primer-BLAST (See Table S3). Assay primer pairs were designed to span a large intron with binding sites on adjacent exons with similar melting points (Tm = 60) and amplicon sizes ranging from 70 to 120 nucleotides. Tata-binding protein (*Tbp*) or ribosomal protein L32 (*Rpl32*) mRNAs were verified to be stably expressed among groups and treatments and were used as reference genes (depending on tissue). Gene expression data were normalized to the expression of *Tbp* or *Rpl32* (2^-ΔCq^) and presented relative to expression levels in the control group.

### Immunoblotting

Liver and oWAT tissue were added lysis buffer (1% SDS, 10 mM Tris–HCl, 10 mM EDTA containing cOmplete Proteinase Inhibitor Cocktail (#11836170001, Roche)) and phosphatase inhibitors (#P0044, Sigma-Aldrich), added glass beads and homogenized using a Precellys 24 tissue homogenizer (Bertin Instruments). Samples were mixed with Laemmli buffer, heated at 95 °C for 5 min, and loaded into Criterion TGX 4 to 20% gels (Bio-Rad). Gels were transferred to nitrocellulose using Trans-Blot Turbo RTA Transfer kit (Bio-Rad), and membranes were stained with Ponceau S to determine the total protein loaded in each lane. Primary antibodies used are as follows: rabbit anti-Atf4 (#11815, Cell Signaling Technology), mouse anti-CHOP (#2895, Cell Signaling Technology), rabbit anti-eIF2α (#9722, Cell Signaling Technology), rabbit anti-Phospho-eIF2α (#3398, Cell Signaling Technology), rabbit anti-Pdha1 (#3205, Cell Signaling Technology), rabbit anti-Phospho Pdha1 (#ABS-194, Milipore), rabbit anti-PERK (#3179, Cell Signaling Technology), rabbit anti-Pkm (#3106, Cell Signaling Technology), rabbit anti-Plin1 antibody from the Londos laboratory (60), guinea pig anti-Plin2 (#GP40, Progen), guinea pig anti-Plin3 (#GP36, Progen), rabbit anti-Plin4 antibody (51), guinea pig anti-Plin5 (#GP31, Progen), rabbit anti-Pygm (#ab81901, Abcam), and mouse anti-beta actin (#A-2228, Sigma). Secondary antibodies used are as follows: goat anti-rabbit IgG (#111-035-144), goat anti-mouse IgG (#115-005-003), donkey anti-guinea pig IgG (#706-035-148) from Jackson ImmunoResearch. Band intensities were quantified using ImageJ software. Immunoblot signals were normalized to total Ponceau stain or beta-actin levels (liver) in the corresponding lane. Data are shown relative to signal intensities for the *Plin4*^+/+^ CD group.

### Detailed lipidomics analysis

Lipids were extracted from plasma using ice-cold isopropanol (1:3, v/v), vortexed for 10 s, and centrifuged at 21,000×*g* for 30 min at 4 °C. Liver tissue (20 mg) was extracted in 200 μl of ice-cold isopropanol and homogenized with a tissue homogenizer (Precellys Evolution, Bertin) using 2.8 mm ceramic beads (two cycles: 20 s at 6800 rpm with a 20 s pause). Homogenates were centrifuged at 21,000×*g* for 30 min at 4 °C. Supernatants were transferred into glass vials, stored in an autosampler at 4 °C, and analyzed immediately. An aliquot of the lipid extracts of every liver sample was also diluted 1:9 using isopropanol and additionally subjected to lipidomics analysis to ensure a linear response for highly abundant lipids. A pooled quality control (PQC) sample was prepared from aliquots of plasma and liver extracts separately and injected at every fifth run for signal correction.

Global lipidomics analysis was performed using a Vanquish Horizon UHPLC coupled to a Fusion Orbitrap Tribrid MS (Thermo Fisher Scientific). Lipids were separated on an Accucore C30 column (150 mm × 2.1 mm, 2.6 μm particle size; Thermo Fisher Scientific). The composition of mobile phases, gradient, column conditions, and mass spectrometer parameters have been described previously (61). Data acquisition was performed with Xcalibur software (version 4.3, Thermo Fisher Scientific). Data were processed with Compound Discoverer (version 3.3.3.200, Thermo Fisher Scientific) for retention time alignment, feature detection and grouping, and signal normalization using the SERRF algorithm (systematic error removal using random forest) based on PQC samples. Features with a coefficient of variation > 30% in PQC replicates were excluded, as well as those detected in processing blanks. LipidSearch software (version 5.1.6, Thermo Fisher Scientific) was used for lipid identification *via* fragmentation spectra matching. Only lipids with grade A-C levels annotation were considered in data analysis, and a manual verification of the included lipids was performed. Lipids, for which there were multiple isomeric species detected, were labeled in lowercase letters (a, b, c, …) after their annotation.

Statistical analyses were performed in R software (version 4.3.1) with *readr*, *dplyr*, and *tidyr* packages. Lipidomics data were obtained using an untargeted, semiquantitative platform. As such, lipid abundances represent relative intensities rather than absolute concentrations. Statistical comparisons were performed independently for each lipid species across experimental groups. To identify group-wise differences, we applied two-sided Welch *t* test and adjusted for multiple comparisons using the Benjamini–Hochberg false discovery rate procedure. Statistical significance was defined using adjusted *p*-values (*p* < 0.15).

The raw and processed lipidomics data were uploaded to the MassIVE repository under the code MSV000099281 and can be accessed under: https://doi.org/10.25345/C5VD6PJ1M.

### Determination of TAG, cholesterol levels, and AST and ALT

TAG and cholesterol levels in plasma, lysates of liver, and oWAT were measured using enzymatic detection kits for TAG (#T75321L, Pointe Scientific, Canton), total cholesterol (#C75101L, Pointe Scientific), AST (#A7561, Pointe Scientific), and ALT (#A7526, Pointe Scientific) following the manufacturer’s instructions.

### Quantification of adipocyte cell size and crown-like structures

Frozen oWAT tissue was fixed in 4% paraformaldehyde solution, embedded in paraffin, and prepared as 5 μm thick sections. Sections selected for imaging were separated by at least 20 sequential cuts (>100 μm between sections) to exclude that adipocytes were counted on multiple sections.

#### Quantification of adipocyte size

Tissue sections were stained with H&E. Representative pictures were taken at 20x magnification using an Olympus BX61 microscope (Olympus). The adipocyte cell size (diameter) was measured using the Adiposoft plugin (62) for ImageJ (U. S. National Institutes of Health) and recorded as μm per adipocyte. Measured diameters below 20 μm were considered to represent nonadipocytes and were excluded from analysis.

#### Quantification of crown-like structures

Sections were deparaffinized with EnVisionTM FLEX high PH retrieval solution (#K8005, Dako, Glostrup) and treated with the EnVision Peroxidase-Blocking reagent (#SM801, Dako) for 5 min to block endogenous peroxidase activity. Sections were subsequently incubated overnight with anti-mouse/human Mac2 (Galectin-3) antibody (#125401, BioLegend), followed by incubation with a horseradish peroxidase–linked secondary anti-mouse antibody (#K5007, Agilent) for 1 h at room temperature. Detection of horseradish peroxidase was done using 3′3 diaminobenzidine tetrahydrochloride, and sections were then stained for 10 min with Hagen’s hematoxylin. Pictures of whole tissue sections were taken using AxioScan Z1 (Carl Zeiss AG). The number of crown-like structures was manually counted and normalized to tissue area (mm^2^) using ZEN Blue software v2.6 (Carl Zeiss AG).

### Statistics

All data were analyzed with Prism 10 (GraphPad Software) or R software, version 4.3.1. For lipidomics analysis, an adjusted *p*-value (<0.15) was defined as threshold for significance. For all other analyses, *p* < 0.05 was defined as significant. Data in graphs are presented with the measured value for each individual (dots/squares), columns indicate the mean values for groups, and error bars indicate ± 95% confidence intervals, unless otherwise stated.

## Data availability

Data that support the findings of this study are available from the corresponding author upon reasonable request. The raw and processed lipidomics data can be accessed in the MassIVE repository under the code MSV000099281 (https://doi.org/10.25345/C5VD6PJ1M).

## Supporting information

This article contains supporting information.

## Conflict of interests

The authors declare that they have no conflicts of interest with the contents of this article.
