# Supplementary material for: Elevated adipose inflammation, but reduced hepatic triacylglycerol storage in diet-induced obese Plin4−/− mice
Source: J Biol Chem. 2025 Dec 13;302(2):111043. doi: 10.1016/j.jbc.2025.111043 (PMC12809094; doi:10.1016/j.jbc.2025.111043)
Supplement: Supplementary Material 1 [file mmc1.docx]

*Supporting Information*

**Elevated adipose inflammation, but reduced hepatic triacylglycerol storage in diet-induced obese *Plin4*^−/−^ mice**

Atanaska Ivanova Doncheva^1^, Ryoko Higa^1^, Prabhat Khanal^1,^, Martine Villemo Øksenvåg Ingebrigtsen^1^, Yuchuan Li^1^, Shrikant Kolan^1^, Pratibha Kolan^1^, Ales Kvasnicka^2,3^, Ingunn Jermstad^1,4^, Shaista Khan^1,4^, Bjørn Steen Skålhegg^1^, Svein Olav Kolset^1^, Hilde Nebb^1^, Marit Hjorth^1^, Frode Amador Norheim^1^, and Knut Tomas Dalen^1,4^*

*^1^Department of Nutrition, Institute of Basic Medical Sciences, Faculty of Medicine, University of Oslo, Norway.*

*^2^ Department of Medical Biochemistry, Oslo University Hospital, Oslo, Norway.*

*^3^ Core Facility for Global Metabolomics and Lipidomics, Faculty of Medicine, University of Oslo, Oslo, Norway.*

*^4^The Norwegian Transgenic Center, Institute of Basic Medical Sciences, University of Oslo, Norway.*

**Running title**: *Plin4 and obesity*

Correspondence to:

*Knut Tomas Dalen: Department of Nutrition, Institute of Basic Medical Sciences, Faculty of Medicine, University of Oslo, P.O. Box 1046 Blindern, N-0316 Oslo, Norway; Phone: +47-22851515; e-mail: k.t.dalen@medisin.uio.no

**
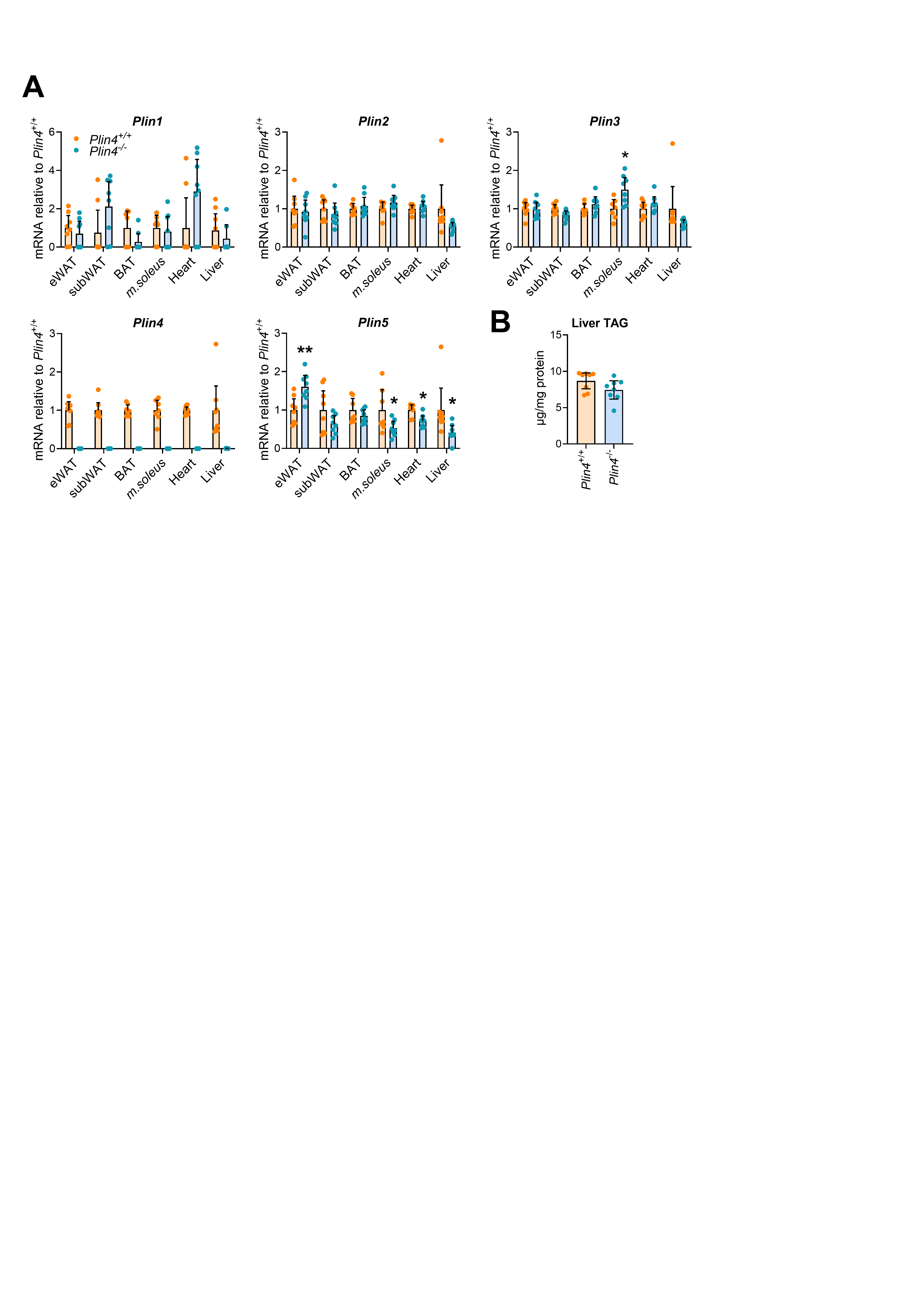
Figure S1**

**Figure S1. Perilipin expression and hepatic TAG levels in male *Plin4*^−/−^ mice fed chow diet**

Male Plin4^+/+^ and Plin4^−/−^ mice (15 weeks of age) were housed with *ad libitum* access to chow diet. Plin mRNA expression levels were determined in selected tissues with RT-qPCR. **A)** Gene expression of *Plin1*, *Plin2*, *Plin3*, *Plin4*, and *Plin*5 mRNAs in epidydimal white adipose tissue (eWAT), subcutaneous white adipose tissue (subWAT), brown adipose tissue (BAT), muscle soleus (m.soleus), heart and liver. Gene expression levels were normalized to expression of TATA-binding protein (*Tbp*) and is shown relative to expression levels in *Plin4*^+/+^ mice. **B)** Liver triacylglycerides (TAG) levels. Statistical testing was done with a t-test (n=8 per group). *P<0.05 and **P<0.01 indicate differences between *Plin4*^+/+^ and Plin4^−/−^ mice for the given tissue. Data are presented as means ± 95% confidence interval.

**
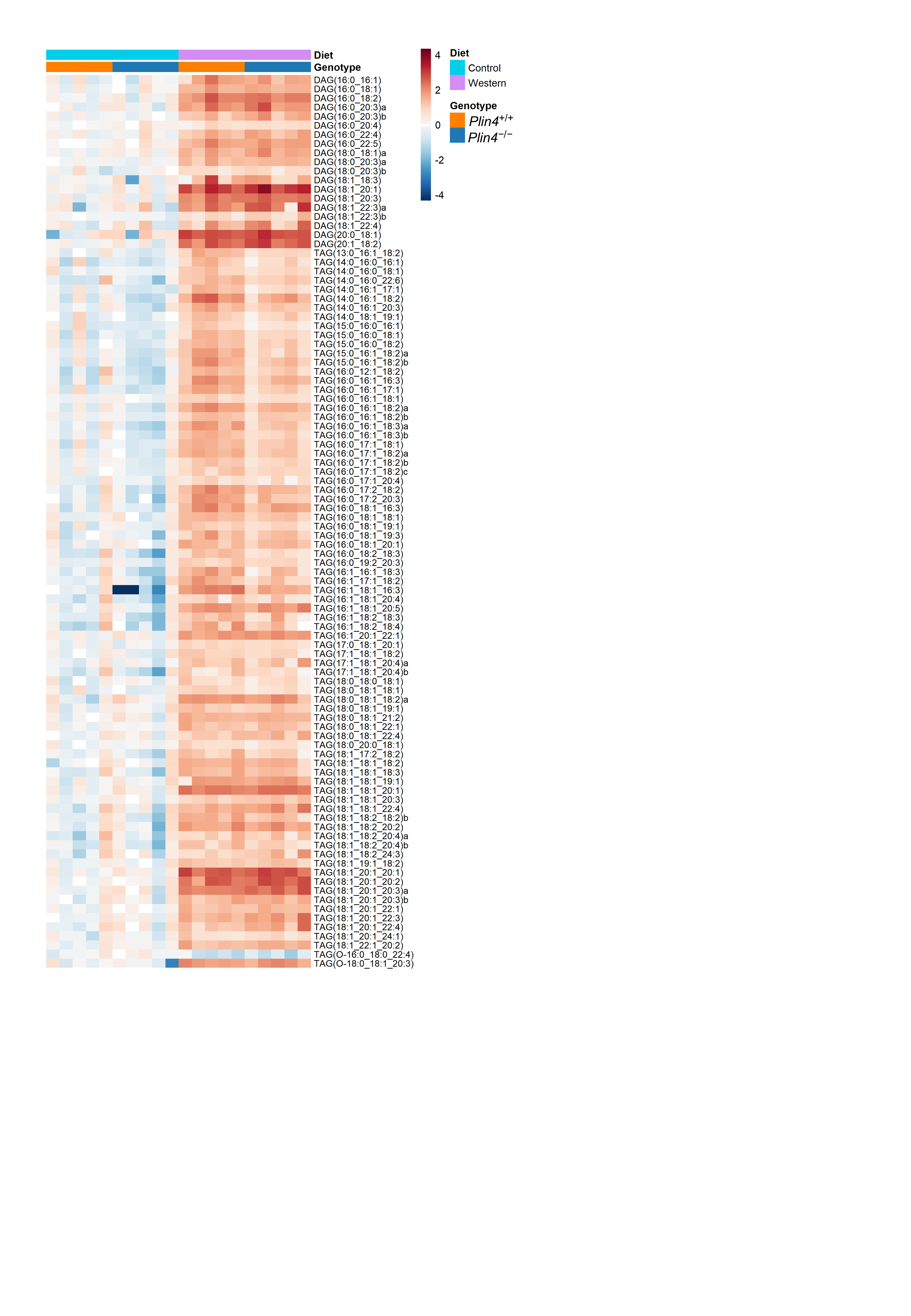
Figure S2**

**Figure S2. Hepatic triacylglycerides (TAG) and diacylglycerides (DAG) levels**

Heatmap showing hepatic DAG and TAG species that are significantly altered between CD and WD in both *Plin4*^+/+^ and *Plin4*^−/−^ (adjusted p < 0.15), with values scaled as log₂ fold change relative to the average levels in the *Plin4*^+/+^ CD group for each lipid species.

**
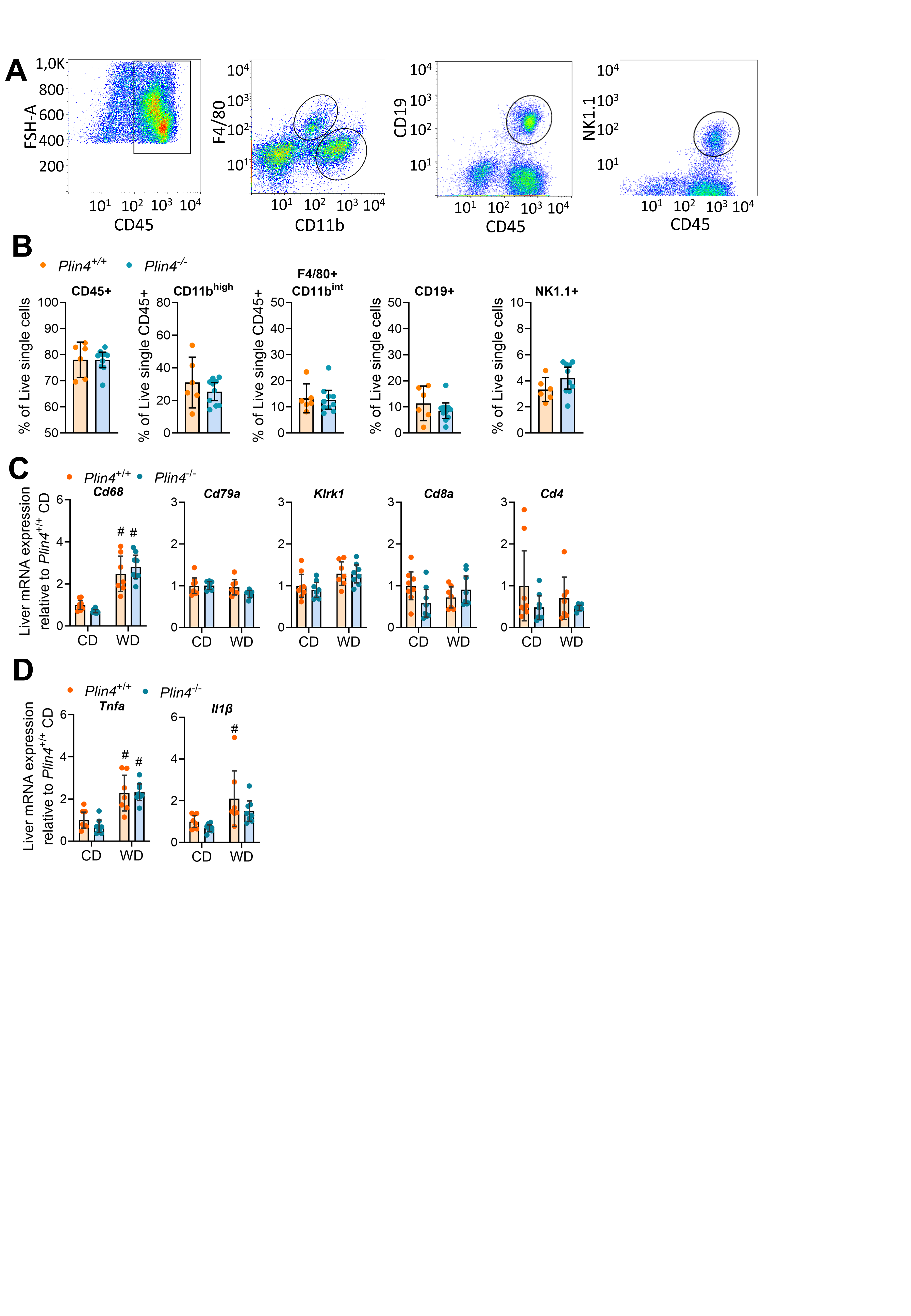
Figure S3**

**Figure S3. Immune cell populations and expression on immune cell markers in *Plin4*^+/+^ and *Plin4*^−/−^ mice fed WD**

Analysis of hepatic immune cells and expression of inflammatory markers in female *Plin4*^+/+^ and *Plin4*^−/−^ mice fed control diet (CD) and Western diet (WD) for 45 weeks. **A)** Gating strategies used for flow cytometry analysis of immune cell suspension. Plots are shown as representative examples of the gating strategies used to detect various immune cell populations. **B)** Quantification of CD45 (total immune cells), CD11b^high^ (monocytes), F4/80^high^/CD11b^intermittent^ (macrophages), CD19 (B cells), and NK1.1 (natural killer cells) cells in the livers of mice fed WD. Student t-test was used to compare *Plin4*^+/+^ (n=6) and *Plin4*^−/−^ (n=10) mice. **C)** Expression of various immune cell markers in mice fed CD and WD: *Cd68*, *Cd79a*, *Klrk1*, *Cd8a and* *Cd4* mRNAs. **D)** Expression of mRNAs representing immune cell markers in mice fed CD and WD: *Tnfa* and *Il1b* mRNAs. Data are shown relative to expression in *Plin4*^+/+^ mice fed CD. *Plin4*^+/+^ CD (n=8), *Plin4*^−/−^ CD (n=8), *Plin4*^+/+^ WD (n=7), and *Plin4*^−/−^ WD (n=8). Statistical testing was performed with two-way ANOVA and the Šídák's multiple comparisons test. # designates differences between diets for each genotype (p<0.05). Data in graphs are shown as means ± 95% confidence interval.

**
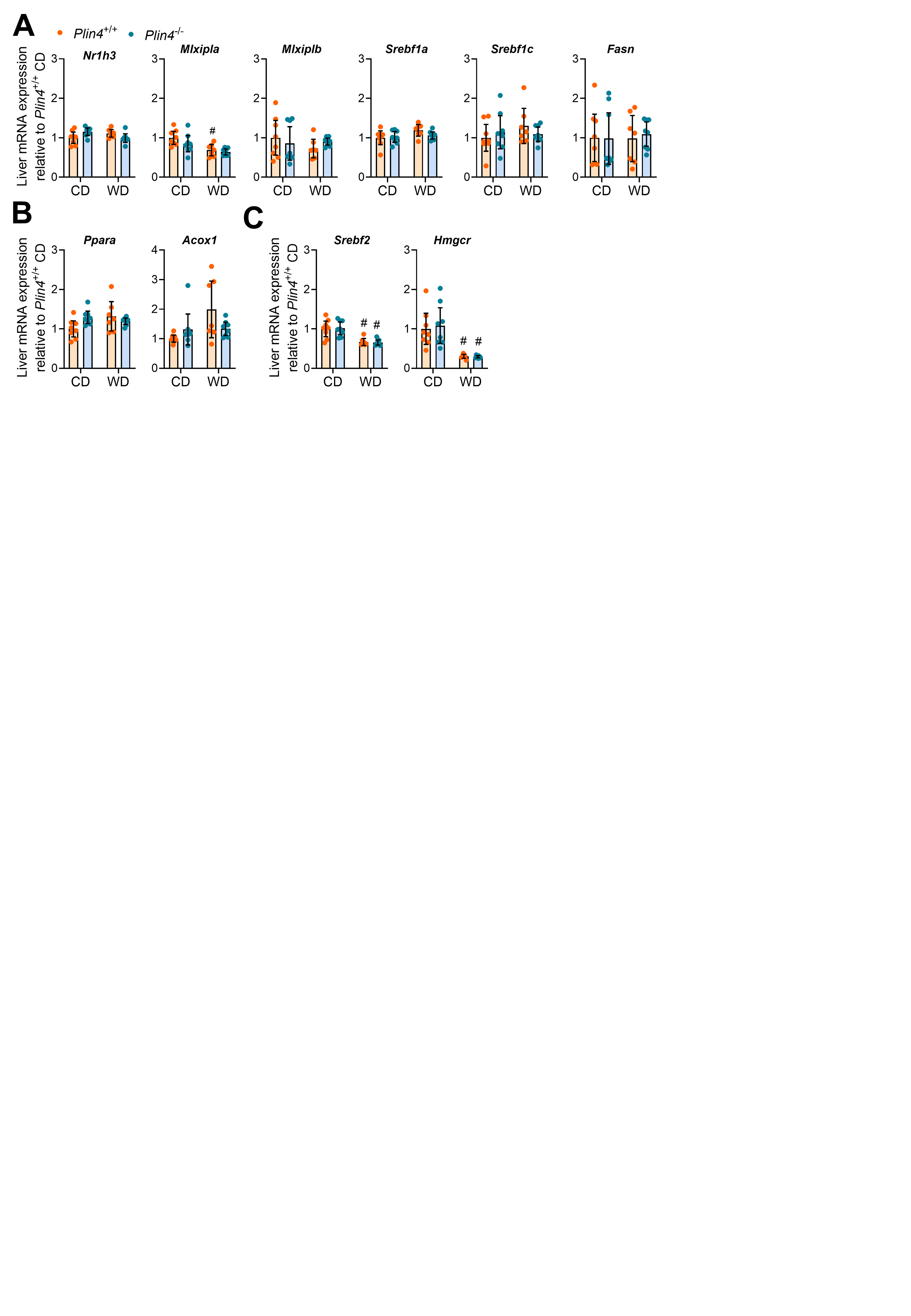
Figure S4**

**Figure S4. Hepatic gene expression in mice fed CD and WD**

Female *Plin4*^+/+^ and *Plin4*^−/−^ mice (10-weeks old) were fed control diet (CD) or Western diet (WD) for 45 weeks. *Plin4*^+/+^ CD (n=8), *Plin4*^−/−^ CD (n=8), *Plin4*^+/+^ WD (n=7), and *Plin4*^−/−^ WD (n=8). RNA was isolated from whole liver and subjected to RT-qPCR analysis. Data was normalized to the expression of TATA-binding protein (*Tbp*) and is presented relative to expression in *Plin4*^+/+^ mice fed CD. **A)** Expression of mRNAs involved in lipogenesis: *Nr1h3*, *Mlxipla*, *Mlxiplb*, *Srebf1a*, *Srebf1c*, and *Fasn*. **B)** Expression of mRNAs involved in β-oxidation: *Ppara* and *Acox1*. **C)** Expression of mRNAs involved in cholesterol synthesis: *Srebf2* and *Hmgcr*. Statistical testing was performed with two-way ANOVA and the Šídák's multiple comparisons test. # indicates differences between diets for each genotype. Data are shown as means ± 95% confidence interval.

**
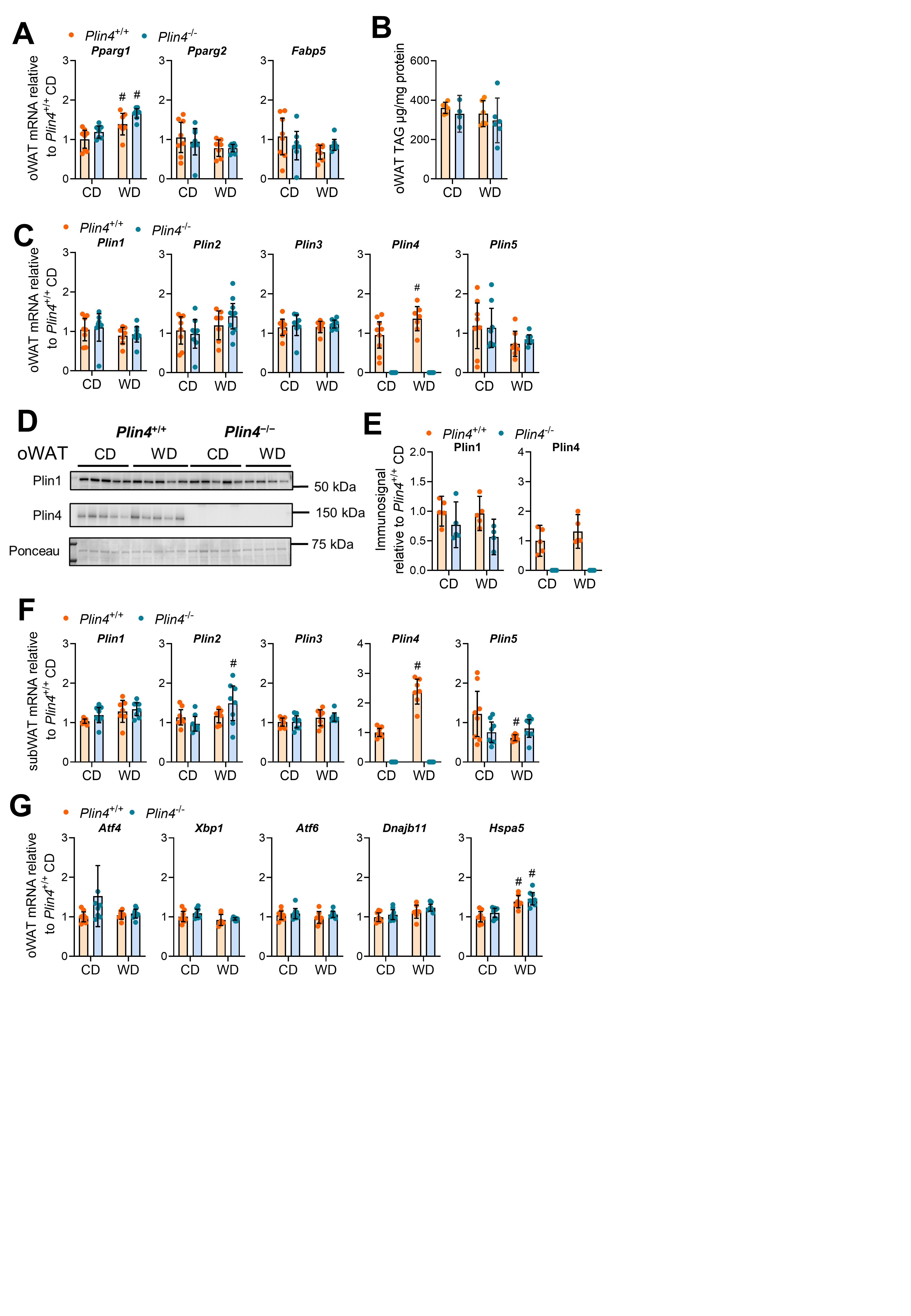
Figure S5**

**Figure S5. Ovarian and subcutaneous white adipose tissue (oWAT) gene and protein expression in mice fed CD and WD**

Female *Plin4*^+/+^ and *Plin4*^−/−^ mice (10-week-old) were fed control diet (CD) or Western diet (WD) for 45 weeks. *Plin4*^+/+^ CD (n=8), *Plin4*^−/−^ CD (n=8), *Plin4*^+/+^ WD (n=7), and *Plin4*^−/−^ WD (n=8). mRNA data was normalized to the expression of TATA-binding protein (*Tbp*) and is presented relative to expression in *Plin4*^+/+^ mice fed CD. **A)** Expression of *Pparg1*, *Pparg2* and *Fabp5* mRNAs in ovarian WAT (oWAT), relative to expression in *Plin4*^+/+^ mice fed CD. **B)** TAG levels in oWAT. **C)** Expression of *Plin1*, *Plin2*, *Plin3*, *Plin4*, and *Plin5* mRNAs in oWAT, relative to expression in *Plin4*^+/+^ mice fed CD. **D)** Representative immunoblots of Plin1 and Plin4 protein expression in oWAT. *Plin4*^+/+^ CD (n=5), *Plin4*^+/+^ WD (n=5), *Plin4*^−/−^ CD (n=5), and *Plin4*^−/−^ WD (n=4). **E)** Relative quantification of Plin1 and Plin4 immunosignals normalized to Ponceau staining in oWAT (n=4-5). **F)** Expression of *Plin1*, *Plin2*, *Plin3*, *Plin4*, and *Plin5* mRNAs in subcutaneous WAT (subWAT), relative to expression in *Plin4*^+/+^ mice fed CD (n=7-8). **G)** Expression of mRNAs involved in ER stress response: *Atf4, Xbp1, Atf6, Dnajb1,* and *Hspa5* (n=7-8)*.* Statistical testing was performed with two-way ANOVA and the Šídák's multiple comparisons test. # indicates differences between diets for each genotype. Data are shown as means ± 95% confidence interval.

**Figure S6**

**
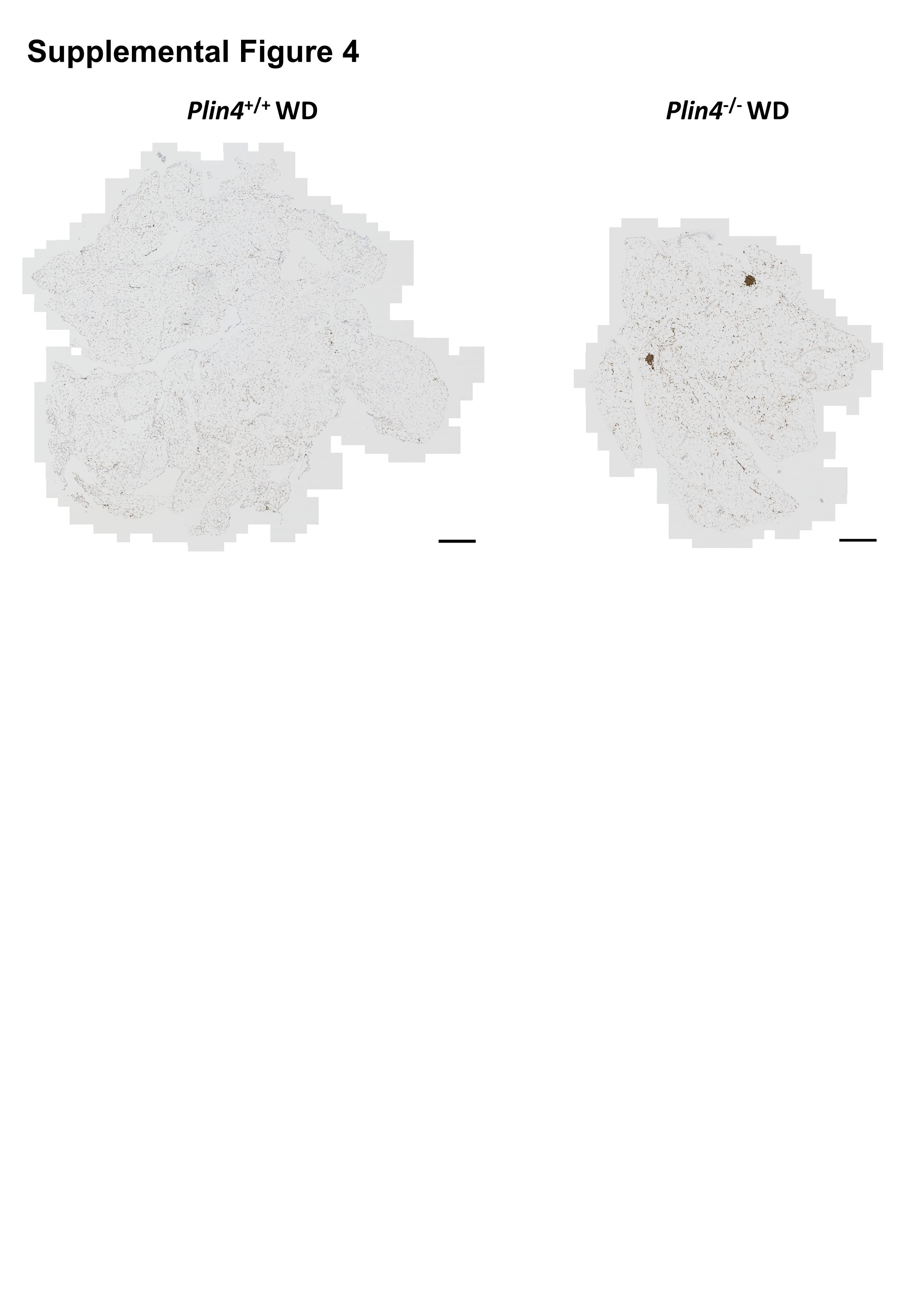
**

**Figure S6. Crown- like structures in oWAT sections**

Analysis of ovarian white adipose tissue (oWAT) from female *Plin4*^+/+^ and *Plin4*^−/−^ mice fed Western diet (WD) for 45 weeks (from 10-weeks to 55 weeks of age). Representative whole-scan images of 5 μm thick oWAT sections from *Plin4*^+/+^ and *Plin4*^−/−^ mice. Sections were stained with Mac2 antibody to visualize crown-like structures (brown ring structures). Scale bar is 1000 μm.

**Table S1. Primers used for cloning**

Primers used for cloning of the *Plin4 Flox-Neo* vector, generation of probes for Southern, and PCR genotyping. The inserted restriction sites to facilitate cloning are underlined.

| **Primers for generation of homology arms to extract the Plin4 genomic sequence** | | | | | |
| --- | --- | --- | --- | --- | --- |
| *Primer name* | | *Inserted RE site* | | *Sequence* | |
| Plin4_5-arm-fw | | *NotI* | | ATAAGCGGCCGCCAGGTTGGATTTCTCCCTCCACTA | |
| Plin4_5-arm-rev | | *HindIII* | | GTCAAGCTTTTAAGTCCCTGTGCCTGCTATGG | |
| Plin4_3-arm-fw | | *HindIII* | | GCTAAGCTTCCCAGGAGGGACTCCCATAGTAA | |
| Plin4_3-arm-rev | | *SpeI* | | TCTACTAGTGACCCCAGGCCTAGAAAATAGGG | |
| **Primers for generation of mini-vector targeting Plin4 intron 2** | | | | | |
| *Primer name* | | *Inserted RE site* | *Sequence* | | |
| Plin4-intron2_5-arm-fw | | *NotI* | ATAAGCGGCCGCTTAGTCACTGGGCCTTCAACAGG | | |
| Plin4-intron2_5-arm-rev | | *EcoRI, NheI* | GTCGAATTCGCTAGCGCAGAGTAGCCCTCTGTTCTCTT | | |
| Plin4-intron2_3-arm-fw | | *BamHI* | ATAGGATCCCAGGGACCCTGGGAAGGCAAC | | |
| Plin4-intron2_3-arm-rev | | *SalI* | GTCGTCGACCACCCCAGTTGAACCTCAGAATG | | |
| **Primers for generation of mini-vector targeting Plin4 intron 6** | | | | | |
| *Primer name* | | *Inserted RE site* | *Sequence* | | |
| Plin4-intron6_5-arm-fw | | *NotI* | ATAAGCGGCCGCCACCACCAGAGACACCCAGTCTT | | |
| Plin4-intron6_5-arm-rev | | *EcoRI* | GTCGAATTCCTCTAGGGACCCGTGATAGGGAA | | |
| Plin4-intron6_3-arm-fw | | *BamHI, NheI, SwaI* | ATAGGATCCGCTAGC*ATTTAAAT*CCTGGGCTTTGGTGAGACCCTAT | | |
| Plin4-intron6_3-arm-rev | | *SalI* | GTCGTCGACGTGGGTGGAATATTGGGGAAAGA | | |
| **Primers used to generate probes for Southern screening** | | | | | |
| *Primer name* | | *Sequence* | | | *Fragment size (bp)* |
| Plin4_5-probe-fw | | TTAAAGGCCCGGTTTCATATTGC | | | 498 bp |
| Plin4_5-probe-rev | | CTTCTGAGGCCCACTTCCTTTGT | | |  |
| Plin4_3-probe-fw | | AGCAGCTAGACAAACCCCCACTC | | | 413 bp |
| Plin4_3-probe-rev | | TTCAACCACAGAAAGCTGCACTG | | |  |
| **Primers used for PCR based genotyping** | | | | | |
| *Primer name* | *Sequence* | | | | *Obtained PCR fragment size (bp)* |
| Plin4 WT and Plin4-flox-Neo | | | | | |
| Plin4-scr1 | CTCTGAGGGACCCTTTACCCTGA | | | | Plin4-WT: 323 bp |
| Plin4-scr2 | CTCAAGGTCTGTGTGGGGTGAGT | | | | Plin4-KO: 434 bp |
| Plin4-scr3 | TGAGTTGAAAGGGCTGGGTGATA | | | | Plin4-WT: 405 bp |
| Plin4-scr4 | TGACCAGTTGGAGGAATCCAGAG | | | | Plin4-FRT-Neo-FRT cassette present: >2317 kb |
| Plin4-scr5 | AATGAGGAAATTGCATCGCATTG | | | | Plin4-FRT-Neo-FRT cassette present: 608 bp |
| Plin4-scr4 | CTCTGAGGGACCCTTTACCCTGA | | | | Plin4-FRT sites recombined: no PCR product |
| FRT sites recombined (floxed) | | | | | |
| Plin4-scr3 | (see above) | | | | Plin4- FRT-Neo-FRT cassette present:> 2317 kb |
| Plin4-scr4 | (see above) | | | | Plin4-flox: 523 bp |
| LoxP sites recombined (null allele) | | | | | |
| Plin4-scr1 | (see above) | | | | Plin4-WT: > 6.9 kb |
| Plin4-scr4 | (see above) | | | | Plin4-KO: 676 bp |
| Cre-fw | ACCAGGTTCGTTCACTCATGGAA | | | |  |
| Cre-rev | CAGACCAGGCCAGGTATCTCTGA | | | |  |
| Flp-fw | CGTGGCCAGGACAACGTATACTC | | | |  |
| Flp-rev | GCTGCCACTCCTCAATTGGATTA | | | |  |

*Abbreviations: fw, forward; KO, knock out; Neo, neomycin; RE, restriction enzyme; rev, reverse; scr, screening; and WT, wild type.*

**Table S3. Primers used for RT-qPCR**

Primers were designed using Primer-BLAST with standardized settings. All assay primer pairs are designed to span a large intron and bind to adjacent exons, have similar melting points (Tm=60±2°C, with max Tm difference 2°C), and generate amplicon sizes ranging from 70 to 120 nucleotides. Primers are designed to amplify all known transcript isoforms for the listed genes, unless isoform discrimination is intended (e.g., *Srebf1a* and *Srebf1c*).

| Gene name | Accession | Forward primer | Reverse primer | Product size | Intron length |
| --- | --- | --- | --- | --- | --- |
| *Acox1* | NM_015729.3 | AATCTGGAGATCACGGGCACTT | GTCTTGGGGTCATATGTGGCAG | 95 | 1443 |
| *Adgre1* | XM_006523601.1 | TGTACGTGCAACTCAGGACT | TCCTGGAGCACTCATCCACA | 94 | 1365 |
| *Atf4* | NM_009716.3 | TGGATGATGGCTTGGCCAGT | TTCTCCAACATCCAATCTGTCCC | 76 | 112 |
| *Atf6* | NM_001081304.1 | TGCCACCAGAAGTATGGGTTC | ACTGACAAGCAGACTCTCGG | 83 | 15287 |
| *Cd4* | NM_013488.3 | CTGGTTCGGCATGACACTCT | GGAAGGAGAACTCCGCTGAC | 92 | 1863 |
| *Cd8a* | NM_009857.1 | GTTCTGTCGTGCCAGTCCTT | TGAGATGTCCCGGTAGGGTG | 94 | 505 |
| *Cd68* | NM_001291058.1 | CTCTAAGGCTACAGGCTGCTC | GACTGGTCACGGTTGCAAGA | 76 | 246 |
| *Cd79a* | NM_008784.3 | TCACTCGGAACAAGGCACAA | ACTCACCGTCATAGTTGCCA | 71 | 670 |
| *Col1a1* | NM_007742.4 | CTGACGCATGGCCAAGAAGAC | CCTCGGGTTTCCACGTCTCA | 88 | 1465 |
| *Dnajb1* | NM_001190804.1 | TGGAAGAAGTGTACGCAGGA | GCATTTCCGTTTGCCAGGAG | 83 | 1658 |
| *Fasn* | NM_007988.3 | CTTCGGCTGCTGTTGGAAGTC | GTGTTCGTTCCTCGGAGTGAG | 80 | 1005 |
| *Hmgcr* | NM_001360165.1 | GCCTTGTGATTGGAGTTGGC | ACACTGACATGCAGCCGAAG | 78 | 2804 |
| *Hk2* | NM_013820.3 | CTTCCCTTGCCAGCAGAACA | TGACCACATCTTCACCCTCG | 95 | 2651 |
| *Hspa5* | NM_022310.3 | CCACGGCTTCCGATAATCAGC | TCCAGTCAGATCAAATGTACCCAG | 104 | 317 |
| *Il1b* | XM_006498795.3 | GCTGAAAGCTCTCCACCTCA | TGTCGTTGCTTGGTTCTCCT | 89 | 1149 |
| *Itgax* | NM_021334.3 | GAGGCTGCAAGCATCATTCG | CGTATTCATGGGAAGGCATCG | 109 | 1107 |
| *Klrk1* | NM_001286018.1 | AGGATCTCCCTTCTCTGCTCA | AGTATCCCACTTTGCTGGCT | 72 | 2568 |
| *Mlxipla* | NM_021455.4 | CCTCTTCGAGTGCTTGAGCC | GGATCTTGTCCCGGCATAGC | 98 | 6411 |
| *Mlxipla* | XM_006504483.2 | GACCCGAGGTCCCAGGAT | CACTTGGGAGAGACCAGCTT | 85 | 23566 |
| *Nr1h3* | NM_001177730.1 | GACTTCAGTTACAACCGGGAAGA | ATTCATGGCTCTGGAGAACTCAAA | 90 | 5129 |
| *Nr1h2* | NM_001285517.1 | GAAGGCGTCCACCATTGAG | AAGTCGTCCTTGCTGTAGGT | 108 | 473 |
| *Pdha1* | NM_008810.3 | CGTGGTTTCTGTCACTTGTGTG | CGTAGGGTTTATGCCAGCCT | 72 | 1833 |
| *Pdk4* | NM_013743.2 | AAGATGCTCTGCGACCAGTA | CAATGTGGATTGGTTGGCCTG | 91 | 1858 |
| *Pkm* | NM_001253883.1 | GAAACAGCCAAGGGGGACTAC | CACAAGCTCTTCAAACAGCAGAC | 108 | 2955 |
| *Plin1* | NM_001113471.1 | ACCTGGAGGAAAAGATCCCG | TTCGAAGGCGGGTAGAGATG | 87 | 1316 |
| *Plin3* | NM_025836.3 | CGAAGCTCAAGCTGCTATGG | TCACCATCCCATACGTGGAAC | 98 | 1147 |
| *Plin4* | NM_020568.3 | ACCAACTCACAGATGGCAGG | AGGCATCTTCACTGCTGGTC | 109 | 1213 |
| *Plin4*Δ7-Δ8 | NM_020568.4 | GCACAACCAGTTCCAAGCCA | GTCACTGCACAGCTTCCCAC | 103 | 614 |
| *Plin5* | NM_001077348.1 | GGTGAAGACACCACCCTAGC | CCACCACTCGATTCACCACA | 115 | 568 |
| *Ppara* | NM_001113418.1 | ACTACGGAGTTCACGCATGT | GTCGTACACCAGCTTCAGCC | 74 | 1710 |
| *Pparg* | NM_001127330.1 | TTGCTGTGGGGATGTCTCAC | AACAGCTTCTCCTTCTCGGC | 70 | 12003 |
| *Ppard* | NM_011145.3 | ACATGGAATGTCGGGTGTGC | CGAGCTTCATGCGGATTGTC | 108 | 1590 |
| *Pygm* | NM_011224.2 | GAGTGGAGGACGTGGAAAGG | CCGAAGCTCAGGAATTCGGT | 77 | 3654 |
| *Rpl32* | NM_172086.2 | CGCAAGTTCCTGGTCCACAA | TGTGAGCAATCTCAGCACAGT | 82 | 1297 |
| *Srgn* | NM_011157.3 | CCTGGTTTGGGGATCTTCAGT | CCCTTCTCCTCGATGCAGTTC | 108 | 9718 |
| *Srebf1a* | NM_001313979.1 | GGCCGAGATGTGCGAACTG | GTTGTTGATGAGCTGGAGCATGT | 70 | 13195 |
| *Srebf1c* | XM_006532716.2 | GGAGCCATGGATTGCACATTT | CAGCATAGGGGGCGTCAAA | 91 | 3078 |
| *Srebf2* | NM_033218.1 | TGACTCTCGGGGACATCGAC | CACCTCCAGGGAAGGAGCTA | 105 | 22247 |
| *Tbp* | NM_013684.3 | AGCCTTCCACCTTATGCTCAG | GCCGTAAGGCATCATTGGACT | 90 | 1145 |
| *Tgfb1* | NM_011577.2 | ATGCCAACTTCTGTCTGGGA | GTTGGTTGTAGAGGGCAAGGA | 84 | 505 |
| *Tnfa* | NM_013693.3 | CCACCACGCTCTTCTGTCTAC | CTGATGAGAGGGAGGCCATT | 85 | 516 |
| *Xbp1* | NM_013842.3 | CTGACGAGGTTCCAGAGGTG | GCAGAGGTGCACATAGTCTGA | 96 | 771 |
|  |  |  |  |  |  |
|  |  |  |  |  |  |
|  |  |  |  |  |  |
|  |  |  |  |  |  |
|  |  |  |  |  |  |
|  |  |  |  |  |  |
|  |  |  |  |  |  |
